# Gait phenotypes in paediatric hereditary spastic paraplegia revealed by dynamic time warping analysis and random forests

**DOI:** 10.1371/journal.pone.0192345

**Published:** 2018-03-08

**Authors:** Irene Pulido-Valdeolivas, David Gómez-Andrés, Juan Andrés Martín-Gonzalo, Irene Rodríguez-Andonaegui, Javier López-López, Samuel Ignacio Pascual-Pascual, Estrella Rausell

**Affiliations:** 1 Department of Anatomy, Histology and Neuroscience, TRADESMA-IdiPaz Universidad Autónoma de Madrid, Madrid, Spain; 2 Center of Neuroimmunology and Service of Neurology, Institute of Biomedical Research "August Pi Sunyer", Hospital Clinic of Barcelona, Universidad de Barcelona, Barcelona, Spain; 3 Child Neurology Unit Hospital Universitari Vall d’Hebron, Vall d’Hebron Institut de Reçerca, Barcelona, Spain; 4 School of Physiotherapy ONCE, Universidad Autónoma de Madrid, Madrid, Spain; 5 Department of Physical Medicine and Rehabilitation, Hospital Universitario Infanta Sofía, San Sebastián de los Reyes, Madrid, Spain; 6 Department of Child Neurology, Hospital Universitario La Paz, TRADESMA-IdiPaz, Universidad Autónoma de Madrid, Madrid, Spain; Boston Children’s Hospital / Harvard Medical School, UNITED STATES

## Abstract

The Hereditary Spastic Paraplegias (HSP) are a group of heterogeneous disorders with a wide spectrum of underlying neural pathology, and hence HSP patients express a variety of gait abnormalities. Classification of these phenotypes may help in monitoring disease progression and personalizing therapies. This is currently managed by measuring values of some kinematic and spatio-temporal parameters at certain moments during the gait cycle, either in the doctor´s surgery room or after very precise measurements produced by instrumental gait analysis (IGA). These methods, however, do not provide information about the whole structure of the gait cycle. Classification of the similarities among time series of IGA measured values of sagittal joint positions throughout the whole gait cycle can be achieved by hierarchical clustering analysis based on multivariate dynamic time warping (DTW). Random forests can estimate which are the most important isolated parameters to predict the classification revealed by DTW, since clinicians need to refer to them in their daily practice. We acquired time series of pelvic, hip, knee, ankle and forefoot sagittal angular positions from 26 HSP and 33 healthy children with an optokinetic IGA system. DTW revealed six gait patterns with different degrees of impairment of walking speed, cadence and gait cycle distribution and related with patient’s age, sex, GMFCS stage, concurrence of polyneuropathy and abnormal visual evoked potentials or corpus callosum. The most important parameters to differentiate patterns were *mean pelvic tilt* and *hip flexion at initial contact*. Longer time of support, decreased values of hip extension and increased knee flexion at initial contact can differentiate the mildest, near to normal HSP gait phenotype and the normal healthy one. Increased values of knee flexion at initial contact and delayed peak of knee flexion are important factors to distinguish GMFCS stages I from II-III and concurrence of polyneuropathy.

## Introduction

The Hereditary Spastic Paraplegias (HSP) are a diverse, heterogeneous and large group of neurodegenerative and neurodevelopmental diseases, of which the main common feature is the retrograde degeneration of the cortico-spinal and posterior column pathways [1–3]. Patients with uncomplicated forms of HSP exhibit a combination of pyramidal syndrome and loss of proprioception, limited to or predominantly affecting the lower limbs. Patients with complicated forms of HSP manifest other associated neurological signs as a result of concomitant lesion of other parts of the nervous system (polyneuropathy, ataxia, mental retardation, epilepsy, dementia, extrapyramidal disorders, etc.)[1–3].

The alteration of gait is one of the most frequent clinical signs in HSP patients[1], and it probably results from the combination of a number of factors such as spasticity, hyper-reflexia, hypertonia, loss of strength and loss of selective control[4–6], impaired proprioception and vibratory sensitivity[7, 8], additional loss of sensation and strength caused by contingent polyneuropathy[9], ataxias, dystonia, and secondary musculoskeletal disorders[1–3]. The quantitative assessment of gait in these patients has been greatly facilitated by techniques of 3D-instrumented gait analysis (IGA) [10]. This powerful tool yields accurate measurements of the multiple joint kinematic events that occur during a gait cycle, i.e., between two heel strikes of the same foot. The first approaches for gait assessment in HSP children consisted in calculations of the deviation from normalcy of a number of isolated kinematic gait cycle parameters [11, 12], on the basis of IGA spot measurements, or averaged across the cycle. Those reported that HSP patients show lower *cadence* (steps per minute), lower *walking speed*, lower *step length*, increased *double support time*, higher values of *mean pelvic tilt*, greater *hip flexion at initial contact* and during *swing phase*, lower *hip extension in stance* phase, increased *knee flexion at initial contact* and decreased *range of knee flexion* than compared to healthy children [11–13]. Quantification and comparison of spot measurements or averaged parameters during the cycle is useful for tracking the progression of the disease and is easily managed by clinicians. However, this approach provides little information regarding gait cycle structure and does not facilitate detection of possible gait phenotypes which might be expressed over the heterogeneous spectrum of underlying HSP pathological factors. That can be better achieved by measuring sagittal angular positions of joints over the whole gait cycle time and comparing them as time series in one or multiple patients. In this context, Wolf et al.[14] first documented the gait heterogeneity of HSP patients using cluster analysis (*k-means*) to classify the results of singular value decomposition, a linear method used to pre-process and simplify kinematic gait data[15]. They defined five types or “patterns” of gait cycles in a joint group of infant and adult patients with HSP, CP and normal subjects, mainly based on knee flexion abnormalities. One of the most useful analytical techniques to compare time series is “Dynamic Time Warping”, (DTW), which has some advantages over singular value decomposition or other linear approaches because it can detect similarities between gait cycles even if those are acquired at different velocities, as commonly occurs after IGA, and it is independent of non-linear variations in the time dimension [16]. Another attractive feature of DTW time series analysis in IGA interpretation is the analysis of data from the whole gait cycle without *a priori* selection of gait parameters, avoiding biases from subjective criteria of selection[17], which makes it a reliable tool for classification. However, results obtained with gait analysis have been classically transferred to clinicians in terms of those isolated values or averaged values of gait cycle parameters that represent easy-to-interpret concepts, such as the angular joint position at a particular moment of the cycle or the range of joint movements during a selected period of the cycle. In order to combine the advantages of these two methods, we propose the use of “random forests” analysis[18]—a machine learning technique that will enable us to link patterns obtained with clustering after DTW analysis of time series with a selection of gait parameters that are important for the classification. Random forests are able to deal with high numbers of predicting variables (even in paradigms with low sample sizes) and non-linear relationships and interactions. They also provide importance measures for each input parameter in the model. These advantages make this technique particularly interesting in phenotyping rare diseases, such as HSP [19–21]. In addition, random forest can be useful to relate gait parameters with clinical features in order to explore the mechanisms underlying the heterogeneous gait abnormalities observed in HSP patients.

Our aim is to investigate sagittal kinematic patterns in the gait of a group of HSP children in order to improve our understanding of the heterogeneity of gait alterations in this disease, and to define those gait parameters and clinical features linked to those patterns so that results can be translated into clinical practice, by means of a new methodological approach that uses a combination of DTW analysis with random forest analysis.

## Methods

### Participants

A group of 26 HSP patients (median age: 7; range: 4–17 years; 11 female/ 15 male) was recruited from the national reference unit of *Hospital Universitario La Paz*, in Madrid, Spain. HSP diagnosis was made by an expert child neurologist (SIPP) based on confirmative genetic tests and/or clinical criteria defined by Harding *et al*. [22] (progressive gait disturbance, signs of pyramidal syndrome such as spasticity in the lower extremities, hyper-reflexia and extensor plantar reflex, and absence of other alternative disorders, with either a positive family history or very suggestive clinical picture). Inclusion criteria were: Gross Motor Function Classification System (GMFCS)[23] stage I-III and ability to walk 10 meters with or without assistance. Patients who had undergone *botulinum* toxin injections in the previous 6 months or orthopaedic surgery in the previous 12 months were excluded, or their inclusion was postponed. A detailed clinical description of every patient included in our study is shown in the supplementary material (S1 Table).

A control group of 33 healthy school-aged children (median age: 7; range 4–16 years, 10 females and 23 males) was also recruited for gait analysis. Inclusion criteria were: adequate schooling, absence of any clinical history of neurological, cardiovascular or systemic diseases, absence of uncorrected visual or hearing impairment, absence of known orthopaedic pathologies in the previous six months and negative results after a screening of unknown orthopaedic pathologies through the exploration protocol “*Scottish Rite Hospital*” [24] and a neurological examination.

Our committee for ethics in human research approved this study and children were all subjected to examination after oral assent and parental written consent.

### 3D-gait analysis methodology

Gait analysis was performed with a Codamotion^®^ system (Charnwood Dynamics Ltd.). This is a system that tracks the position of infrared light emitting markers. The markers were attached to lower limbs following manufacturer instructions (S1 Fig in the supplementary material). According to these positions and anthropometric measures, manufacturer commercial software is able to estimate the position of the centres of different joints and body segments and calculate angular deviations of five joints (pelvis, hip, knee, ankle and forefoot) in three planes (sagittal for flexion and extension, horizontal for abduction and adduction and coronal for rotation) throughout the cycle. After initial training, children were asked to walk from one end to the other of a 15-meter walkway path which allowed five to seven gait cycles per walk, at their natural speed, about 8–12 times. For each subject, up to five left and up to five right gait cycles were selected according to technical quality criteria.

### Gait data extraction and preliminary processing

We processed the kinematic information generated by the commercial system in three sets of data (see supplementary materials, S2 Fig). Firstly, each gait cycle was considered as a multivariate temporal series that corresponds to a matrix of 201 time epochs over the cycle for five different joint movements (pelvis, hip, knee, ankle and forefoot) in sagittal plane (flexion and extension). Secondly, spatio-temporal parameters (*cadence*, *normalized walking speed*, *percentage of time in stance*, *percentage of time in first double support*, *percentage of time in single support* and *percentage of time in double support*) were extracted from each gait cycle (Table 1). Thirdly, 37 right and 37 left isolated or averaged kinematic parameters were selected over the cycle, based on previous literature (Table 2).

**Table 1:** Spatiotemporal parameters that were extracted from each gait cycle.

Number	Spatiotemporal parameters
1	Normalized walking speed
2	Cadence
3	Stance time (%)
4	First double support (%)
5	Single support (%)
6	Second double support(%)

**Table 2:** Kinematic parameters that were extracted from each gait cycle.

Number	Kinematic parameters
1	Mean pelvic tilt
2	Range of pelvic tilt
3	Pelvic rotation at initial contact
4	Mean pelvic rotation
5	Range of pelvic rotation in second double support
6	Range of pelvic rotation in terminal swing
7	Mean pelvic obliquity in stance
8	Hip flexion at initial contact
9	Minimum hip flexion
10	Mean hip flexion in stance
11	Maximum hip flexion in swing
12	Time to maximum hip flexion in swing
13	Range of hip flexion
14	Mean hip abduction in first double support and single support
15	Maximum hip abduction in swing
16	Mean hip rotation in stance
17	Mean hip rotation in swing
18	Knee flexion at initial contact
19	Minimum knee flexion in stance
20	Maximum knee flexion in first double support
21	Maximum knee flexion in single support
22	Minimum knee flexion in single support
23	Maximum knee flexion
24	Time to peak knee flexion
25	Range of knee flexion during second double support and swing
26	Range of knee flexion
27	Dorsiflexion at initial contact
28	Maximum ankle dorsiflexion in stance
29	Minimum ankle dorsiflexion in stance
30	Range of ankle dorsiflexion in stance
31	Mean ankle dorsiflexion in first double support
32	Mean ankle dorsiflexion in single support
33	Mean ankle dorsiflexion in second double support
34	Maximum ankle dorsiflexion in swing
35	Minimum ankle dorsiflexion in swing
36	Range of ankle dorsiflexion in swing
37	Mean foot progression in stance

### Data analysis

#### Pattern detection in HSP gait

We considered a gait pattern as a set of kinematic features that are shared by a group of gait cycles and that make this group different from the rest. We assessed the similarities of kinematic time series in sagittal plane in pelvis, hip, knee, ankle and forefoot joints between gait cycles of HSP patients by means of a multivariate Dynamic Time Warping (DTW) algorithm (R package: DTW). In order to reveal the presence of different gait patterns in HSP, we classified the gait cycles of patients by means of hierarchical clustering analysis using the *Euclidean DTW distance* as dissimilarity measure and *average linkage clustering* as grouping criteria. The DTW algorithm provides dissimilarity measures (Euclidean DTW distances between every pair of gait cycles) and allows measurements of similarities between two temporal sequences that may vary in speed[16]. Then, groups of cycles, patterns, were subjectively defined by visual observation of the dendrogram obtained from hierarchical clustering[25]. A narrative description of the resulting gait patterns was then produced based on the graphic representation of the kinematic plots included in each group. We also investigated the possibility that one subject used one or several sagittal gait patterns.

#### Correlation of sagittal patterns with kinematic parameters

Random forests are machine-learning techniques that are of particular interest in gait analysis [20, 21] as they are able to deal with a high number of predicting variables in situations in which sample size is low or in which parameters are highly correlated with each other. This study applies classification random forests (1000 trees and different numbers of variables tried at each split (1,3,6,12)) to select which kinematic and spatiotemporal parameters (Tables 1 and 2) are relevant to differentiate the gait patterns found by DTW hierarchical clustering.

#### Study of spatio-temporal performance across sagittal patterns

In order to describe the spatio-temporal performance in HSP patients, we generated a *heatmap* with the Z-scores of the spatio-temporal parameters (see Table 1) of all gait cycles, grouped according to the hierarchical classification. To investigate the association of the sagittal patterns with spatio-temporal parameters, we used linear mixed models [26], a regression analysis technique that can be particularly useful when multiple measures from the same subject are taken. Six linear mixed models (one for each spatio-temporal parameter–Table 1) were adjusted for the flexion pattern used in each cycle (fixed effect) and for the subject that performs the cycle (random effect). The significance of the effect of flexion pattern on spatio-temporal parameters was assessed by means of a likelihood ratio test between a simplified model excluding flexion pattern as independent parameter and the saturated model that included it.

#### Analysis of the differences between the least altered HSP gait cycles and those from the healthy control group by means of random forests

Some HSP paediatric patients show milder forms of gait disorder and their gait phenotype (see in Results, HSP flexion Pattern I) is very similar to normal gait. We hypothesized that, in spite of this similarity, machine-learning techniques might detect gait abnormalities that may be overlooked by simpler assessment.

We trained regressive random forests (1000 trees and 1, 14, and 29 variables in each split) with 43 gait parameters (Tables 1 and 2) as input variables to predict whether a gait cycle belongs to healthy children (indicated by value 0), or to the group of HSP cycles that have milder abnormalities (indicated by value 1). We assessed the model goodness of fit with the area under the receiver operating characteristic curve (AUC). The *importance* of the gait parameters for the prediction in the best random forest model was calculated as the difference between prediction error when the input variable is randomly permuted in the out-of-bag data *versus* the prediction error otherwise (VIMP). We represented VIMP of the most *important* gait parameters in a bar chart. In addition, to reveal how these gait parameters distinguish mild cases from normalcy, we plotted the unadjusted marginal effects on the response of the five most important gait parameters in the random forest analysis. These plots show the relationship between the values of the important gait parameters (*X* axis) and the possibility of belonging to the group of HSP cycles with mild gait abnormalities (*Y* axis).

#### Relationships between gait patterns and clinical features

We investigated the possibility that gait patterns were related to other clinical features. We explored: 1) The severity of the motor disorder represented by the GMFCS stage of the patient. 2) The time-course of the disease, represented by the patients´s age, since it is difficult to establish the age of disease onset in paediatric HSP patients as they nearly always start with mild symptoms that could be variably detected by the family and patient’s doctor. 3) Gender. 4) Presence of peripheral nerve disease (polyneuropathy), evidenced by electrophysiological techniques, that we defined by the presence of two of the following criteria: abnormal sensitive nerve action potential in two nerves, decreased CMAP amplitude for two muscles and EMG denervation signs. A clinician with significant experience in paediatric neurophysiology (SIPP) performed all the examinations. The number of nerves and muscles that were explored was determined based on clinical experience. The minimum protocol includes the assessment of nerve conduction velocity and amplitude of two sensory nerves and two motor nerves and the EMG evaluation of two muscles (one proximal—generally *vastus lateralis*—and one distal muscle—generally *extensor digitorum brevis*). 5) Presence of abnormal patterned visual evoked potentials. 6) Detection of thin *corpus callosum* by brain MRI based on subjective assessment by a paediatric neurologist (DGA).

We described the distribution of these clinical features across the different patterns by means of violin plots (for quantitative clinical features) and bar plots (for qualitative clinical features). We also analysed the effect of those clinical features on the gait pattern classification by means of linear models (for quantitative clinical features) and log-linear models (for qualitative clinical features). We used the corresponding clinical feature as dependent variable and the gait pattern as independent variable. We defined the gait pattern as a dummy variable using the most common pattern as reference. We contrasted the absence of effect of the gait pattern on the clinical feature by analysis of the variance. We transformed GMFCS to a binary variable by grouping cycles from patients with GMFCS II and III all together.

#### Correlation between gait parameters and clinical features by means of random forests

In order to understand better how the clinical features influence gait performance, we also looked for gait parameters that were related to each clinical feature by means of regressive random forests (1000 trees and 1, 7, 14, and 28 variables in each split) with 43 gait parameters (Tables 1 and 2) as input variables. The output variables were the following clinical features: age (quantitative parameter), sex (binary parameter), GMFCS, presence of polyneuropathy, abnormal visual evoked potentials (VEP) and presence of thin corpus callosum at brain MRI. We trained a random forest to predict patient’s sex codifying female as value 0 and male as value 1. We transformed GMFCS into a binary parameter using value 0 for patients with GMFCS level I and value 1 for patients with GMFCS level II and III. In the cases of polyneuropathy, visual evoked potentials and presence of thin corpus callosum at brain MRI, we trained the random forest to predict whether a patient would show an abnormality in the clinical feature (indicated by value 1) or not (indicated by value 0).

We assessed the model goodness of fit with the area under the receiver operating characteristic curve (AUC) for binary parameters and Spearman’s *rho* for quantitative parameters. In the best random forest model, we calculated the *importance* of gait parameters for the prediction as the difference between prediction error when the input variable is randomly permuted in the out-of-bag data *versus* the prediction error otherwise (VIMP). We represented VIMP of the most *important* gait parameters in a bar chart.

In the case of the four most important gait parameters for the prediction of age, we adjusted four linear mixed models for gait data from healthy subjects and children with HSP in which the independent variable was the corresponding gait parameter and the dependent variables were condition (healthy versus HSP), age (in years) and the interaction between age and condition. The interaction *beta* coefficient was used to inform how the effect of age was differential between healthy and HSP children. We used a robust linear regression model that computes MM-estimators.

In the case of those important gait parameters for the prediction of the binary clinical features, we used the difference of means for the corresponding gait parameters. By this means, we could show how much effect each clinical feature has in each particular gait abnormality. We calculated the difference between absolute means and the difference between means previously transformed into Z-score using healthy children as reference. We estimated the 95% confidence interval of these differences by means of bias-corrected and accelerated bootstrap.

## Results

### The gait of HSP patients is heterogeneous and can be classified according to sagittal kinematic patterns

**Fig 1:**
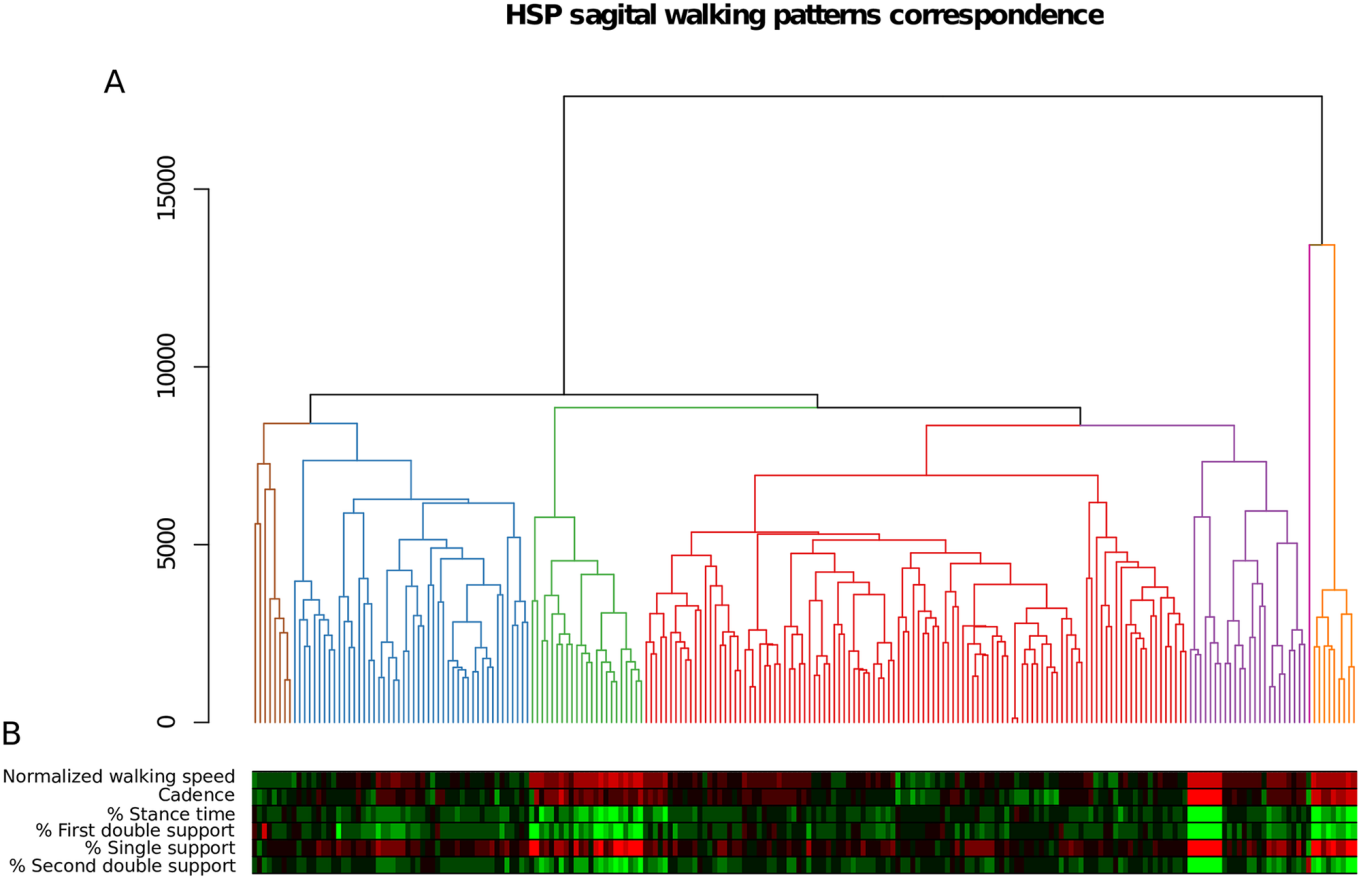
A) Dendrogram obtained from hierarchical clustering analysis of gait cycles of HSP patients. The vertical axis represents the distance DTW. A vertical line that reaches the base indicates each cycle. Horizontal lines that interconnect the vertical ones indicate DTW distances between the cycles. The higher the horizontal lines joining two cycles, the less similar they are. The lower bar indicates whether the classified cycle is from the right limb (red) or the left (green). Six clusters and an “outlier” (pink cycle) are detected. The majority of cycles are grouped within the red and the blue cluster. B) The *heatmap* presents Z-scores (standardized comparison to healthy average value) of the spatio-temporal parameters (rows) of each HSP cycle (columns). Green colour indicates a higher spatio-temporal value in the cycle than in healthy children and red, a lower value. The more intense the colour of the square, the further the value of the cycle is from healthy controls. In comparison to healthy controls, red, blue and brown patterns represent less impaired spatio-temporal performance; while orange, green and part of the cycles from the purple pattern show more altered values.

**Fig 2:**
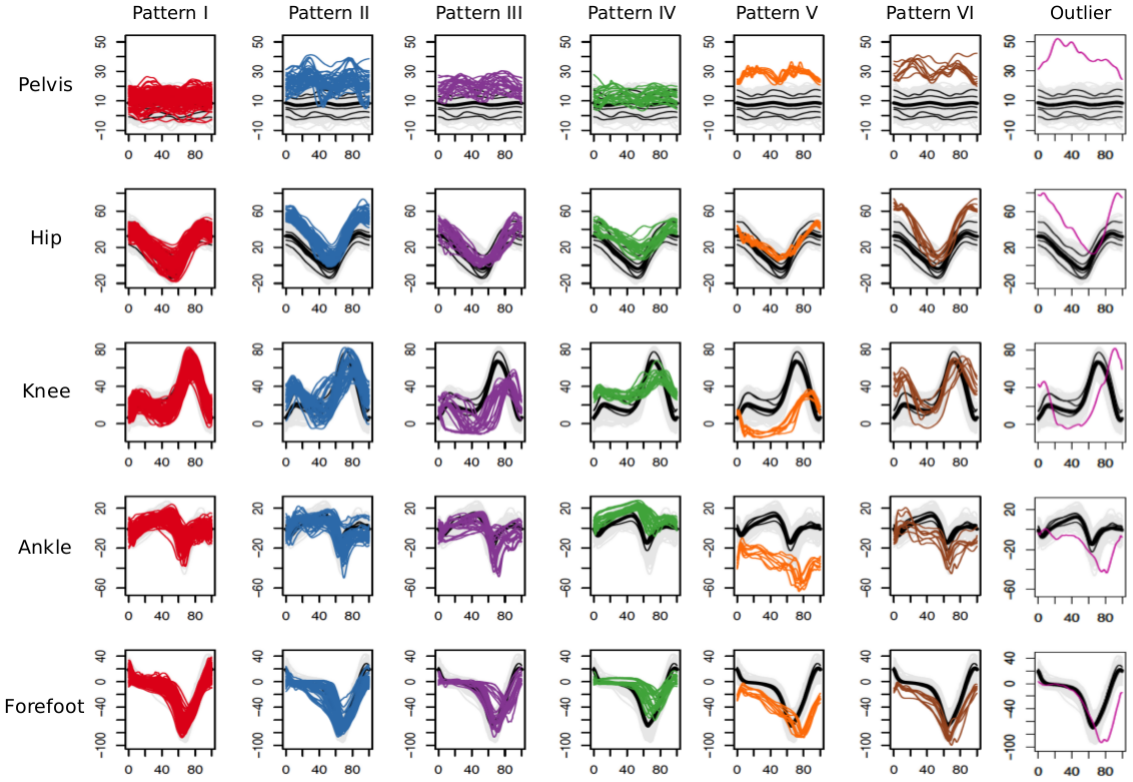
Cumulated kinematic plots of five joints grouped according to seven sagittal patterns yielded by dendrogram in Fig 1. Each column represents a pattern and each row, a joint. The x-axis of each graph corresponds to the percentage of gait cycle. The y-axis represents the joint range in degrees (zero is the neutral position, positive values indicate flexion and negative values, extension). The healthy children’s cycles are depicted in grey lines, their average healthy patterns in black, and the overall healthy average is shown with a thick black line. Patterns I and II (red and blue, respectively) are the most similar to normal. The outlier (pink) corresponds to an “outlier” cycle.

A total of 223 gait cycles from HSP patients were included in the study. Fig 1 shows the results of cluster analysis of those cycles after calculation of DTW distance, that revealed seven groups of HSP cycles. The majority of gait cycles were classified in those clusters represented in red (110 out of 223 total cycles in the classification, 49.3%) and blue (48/223, 21.5%). Fewer cycles were classified in the purple (24/223, 10.8%), green (23/223, 10.3%), orange (9/223, 4%) and brown (8/223, 3.6%) clusters. The pink group has a single cycle considered as an outlier, and corresponds to the single left cycle from subject P26, whose significant left limb gait impairment made it very difficult to obtain technically adequate measures. According to the hierarchical analysis (see dendrogram in the upper part of Fig 2), the red, purple, green, blue and brown clusters share more similarities, while the orange cluster is the most dissimilar and is closely related to the outlier.

Fig 2 shows the kinematic flexion graphs of all cycles for pelvis, hip, knee, ankle and forefoot, separated according the seven groups described in Fig 1. The flexion values of those joints in healthy children are depicted in black overlapped lines. The cycles classified as Pattern I (red in Fig 2) look very similar to normal, but show more anterior *pelvic tilt* throughout the gait cycle than the average of healthy controls, but with few values above the upper limit of the normal range. Most of the cycles classified as Pattern II (blue in Fig 2) show increased anterior *pelvic tilt* during the gait cycle (some cycles even have a “double hump” structure), and hip flexion values throughout the gait cycle are above normal as well; they also show increased values of *knee flexion after initial contact* and delayed *peak value of knee flexion in swing* in some cycles. Ankle and forefoot flexion values are otherwise similar to normal. Gait cycles classified as Pattern III (purple in Fig 2) are characterized by slight anterior *pelvic tilt*, lower than normal values of *hip extension* and very altered behaviour of knee flexion with increased *knee extension in stance*, with some cases of knee *recurvatum* and decreased and delayed *peak value of knee flexion in swing*). Ankle and forefoot flexions are within the normal ranges except from higher and delayed *peak values of plantar flexion* in some cycles. Gait cycles classified as Pattern IV (green in Fig 2) show normal values of *pelvic tilt*, insufficient *hip extension in stance*, increased values of *knee flexion during stance* with decreased and delayed *peak value of knee flexion in swing*, increased ankle dorsiflexion with decreased *peak value of ankle plantar flexion* before swing and decreased and delayed plantar flexion. Gait cycles classified as Pattern V (orange in Fig 2) show increased anterior pelvic tilt with the “double hump” sign, hip flexion in the normal range with decreased hip extension, knee *recurvatum* (that appears quickly after the initial contact), very pronounced plantar flexion in foot and forefoot (*equinus foot*) and a delayed *peak value of plantar flexion* in both joints. Gait cycles classified as Pattern VI (brown in Fig 2) show increased anterior pelvic tilt with “double hump” sign, increased *hip flexion* at initial contact and at the end of swing but normal *hip extension* in stance, increased values of *knee flexion* at initial contact and at the phase of load response with extension in stance, delayed *peak value of knee flexion* and increased *ankle and forefoot plantar flexion*. The cycle that was considered as an outlier (pink in Fig 2) shows a very pronounced anterior pelvic tilt and a very prolonged stance phase that determines delayed peak of hip extension, peak knee flexion, peak ankle and forefoot plantar flexion.

**Fig 3:**
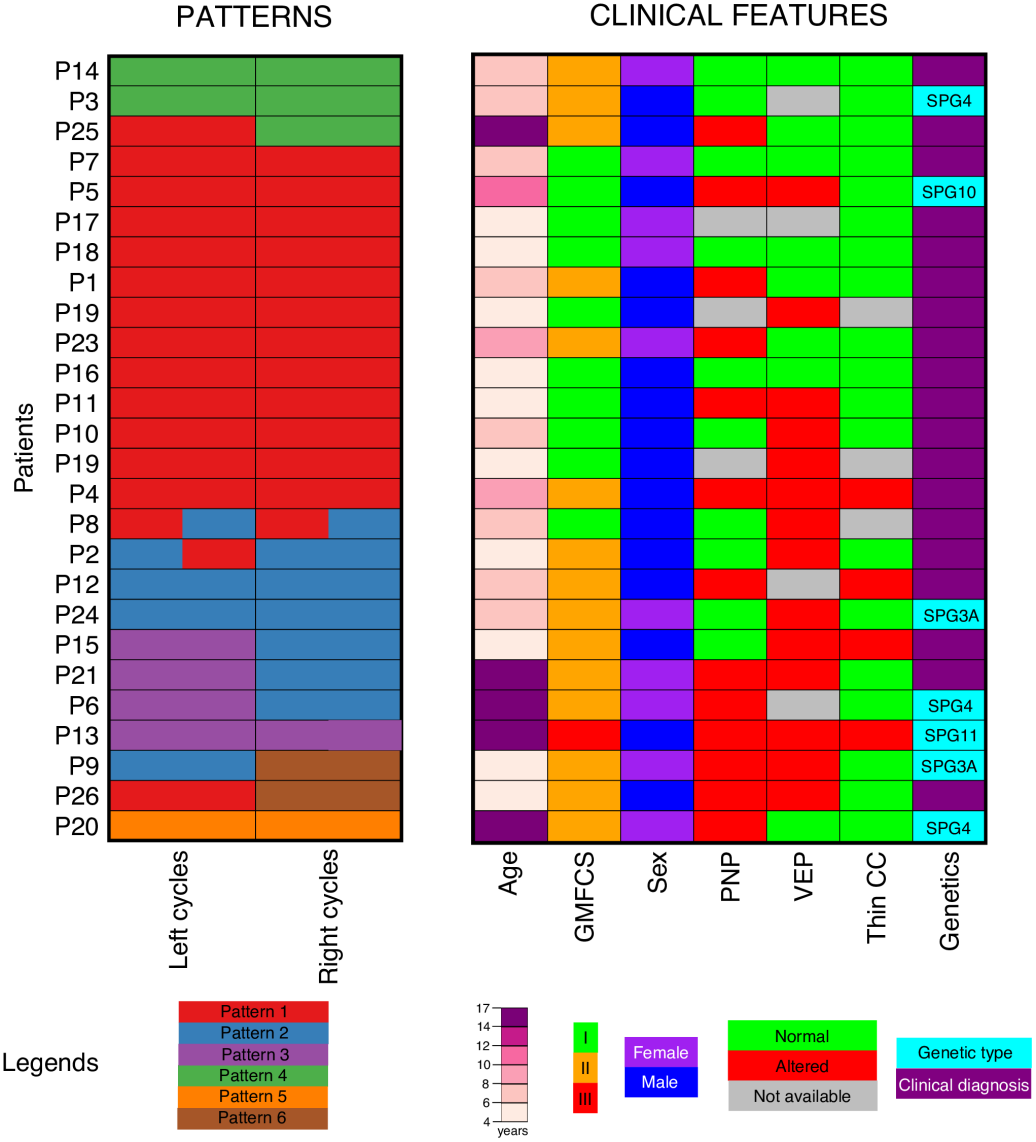
Distribution of sagittal patterns in left and right cycles and clinical features of each patient. Patients were ordered according to the type of gait patterns they use (left) and clinical features were represented with colour scales (right). Notice that clinical features are partially related to the gait phenotype.

The gait patterns used by each patient (left and right cycles) are shown in Fig 3 and S2 Table. 17 out of 26 patients (65.4%) walked using one single pattern. 7 out of 26 patients (27%) used a different pattern in each limb, and 2 out of 26 (7.7%) patients used two patterns in a single limb. This indicates that HSP gait impairment is asymmetric in a considerable number of patients and that some patients can use different sagittal patterns in the same limb.

### Sagittal patterns can be predicted by kinematic parameters

We trained four classification random forests to predict the type of gait pattern in each cycle, based on the values of gait parameters. The best fit was obtained with the random forest with 3 variables tried at each split that shows an overall error rate of 1.35%. It misclassified one cycle of Pattern I as Pattern II (error rate for Pattern I: 0.9%), one cycle of Pattern IV as Pattern I (error rate for Pattern IV: 4.2%) and one cycle of Pattern VI as Pattern II (error rate for Pattern VI: 12.5%).

**Fig 4:**
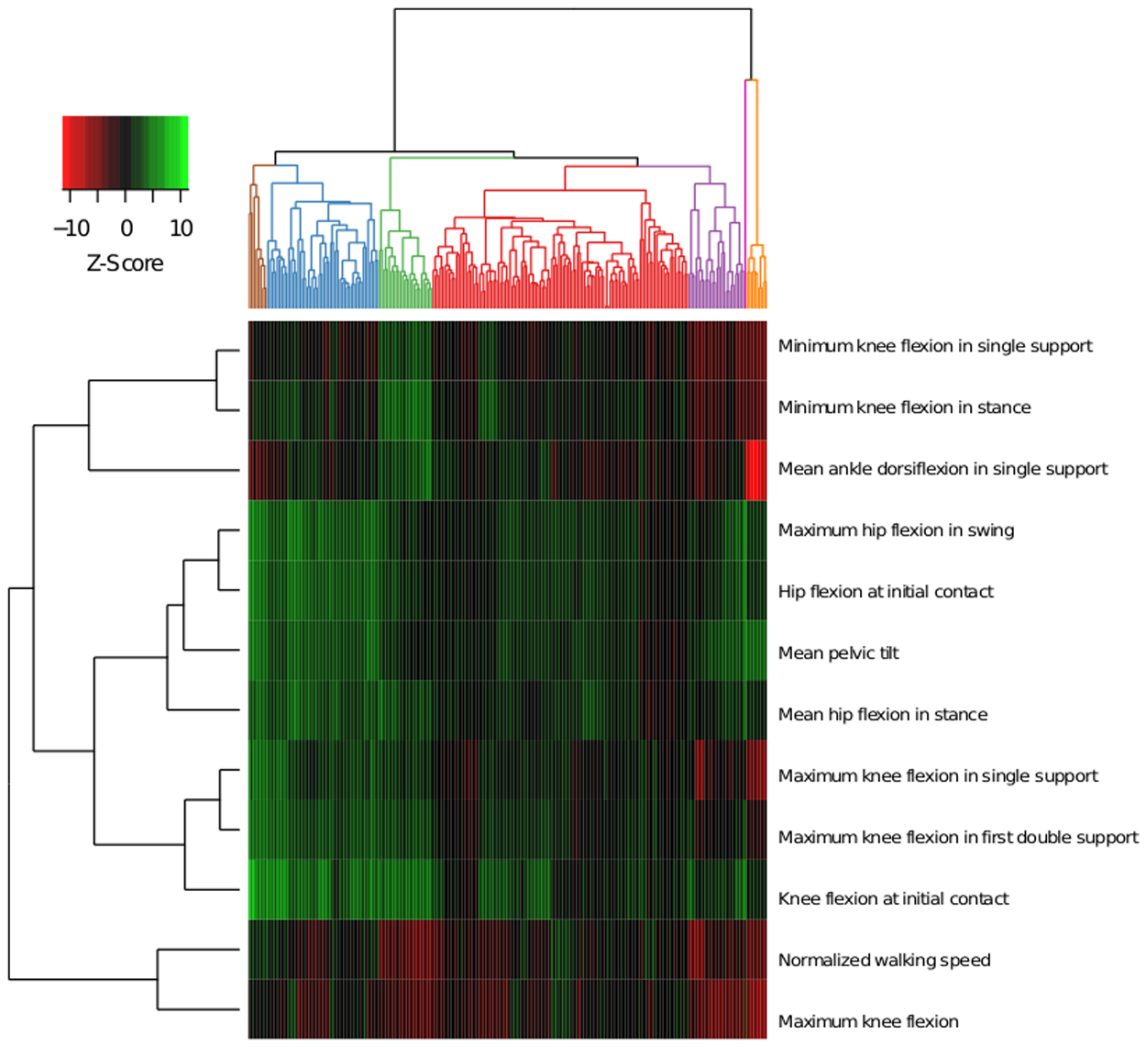
Importance of gait parameters in the classification of gait cycles in sagittal patterns generated by the random forest. It is measured by the mean decrease in accuracy when the variable is out of the bag. The most important are *mean pelvic tilt* and *hip flexion at initial contact*.

The most important gait parameters for classification are shown in Fig 4. The ten most important parameters, by order of importance, are: *mean pelvic tilt*, *hip flexion at initial contact*, *maximum hip flexion in swing*, *maximum knee flexion in first double support*, *mean hip flexion in stance*, *minimum knee flexion in stance*, *knee flexion at initial contact*, *minimum knee flexion in single support*, *maximum knee flexion in single support* and *maximum value of knee flexion*.

**Fig 5:**
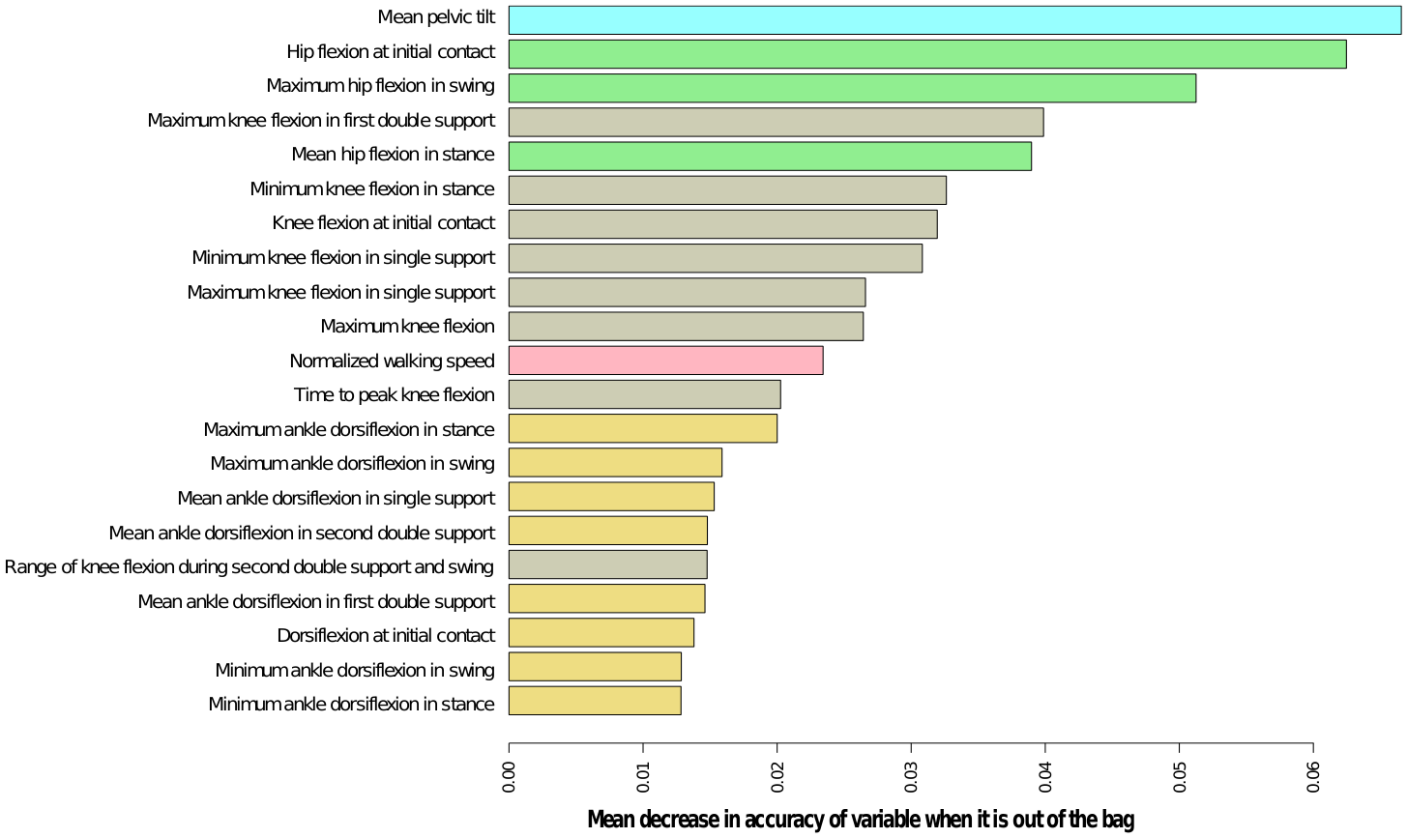
*Heatmap* showing the Z-scores (standardized measure of the distance to healthy average value) of the 12 most important gait parameters to classify cycles into gait patterns according to random forest models. Cycles are represented in columns and ordered following the dendrogram shown in Fig 1. Gait parameters are shown in rows and ordered according to the relationships between relationships with each other (dendrogram on the left). The Z-scores are represented following the colour legend in the top left corner.

To understand which gait parameters are important to distinguish each gait pattern among the rest, we built a *heatmap* (Fig 5) with Z-score of the ten most important parameters (standardized value of distance in relation to the average of healthy children). The important parameters for the model in Pattern I cycles (red) are closer to healthy values (as indicated by a black colour trend). Pattern II cycles (blue) are identified by increased *maximum value of hip flexion in swing* and at *the initial contact*, increased values of *mean hip flexion in stance*, *anterior pelvic tilt* and increased *knee flexion at initial contact*. A subgroup of this group of cycles show decreased *maximum value of knee flexion*. Pattern III cycles (purple) show decreased *minimum value of knee flexion in single support* and stance and reduced *maximum value of knee flexion*. In contrast, Pattern IV cycles (green) show a significant increase of the *knee flexion at initial contact* and higher *minimum value of knee flexion* in stance and single support. Furthermore, they show a decrease in *maximum value of knee flexion* and *normalized walking speed*. Gait cycles classified in Pattern V (orange) show increased anterior *pelvic tilt*, decreased *walking speed* and decreased *mean ankle dorsiflexion in stance* (*equinus* foot). At the knee, the maximum value of flexion is reduced and the decreased values of *maximum knee flexion in single support*, *minimum knee flexion in stance and single support*, and *maximum value of knee flexion* are indicative of knee *recurvatum*. Finally, important kinematic differences from normalcy identify Pattern VI cycles (brown): *increased knee flexion at initial contact*, *increased maximum hip flexion in swing*, *high hip flexion at initial contact*, *anterior mean pelvic tilt and increased maximum knee flexion in single support and in the first double support*.

### Sagittal patterns correlate with different spatio-temporal performances

Fig 1B shows a *heatmap* that illustrates the importance of the spatio-temporal values to classify gait cycles. Additional violin plots of the spatio-temporal values for each cycle are included in the supplementary material (S3 Fig). Cycles in Patterns I (red), II (blue) and VI (brown) show slight differences from normalcy in the spatio-temporal parameters, and those are reflected mainly in increased stance times and decreased percentage of single support. In contrast, spatio-temporal parameters of cycles from Pattern IV (green), V (orange) and part of Pattern III (purple) are far from normal values. These patterns reflect a severe decline in *normalized walking speed*, *cadence and percentage of cycle in single support and stance*, *in first double support and second double support*. Distributions of spatio-temporal parameter values corresponding to each pattern are shown in the supplementary materials.

According to linear mixed models, walking with one of the seven identified HSP flexion patterns significantly affects *cadence* (χ^2^(5) = 11.85, p = 0.03), *first double support time* (χ^2^(5) = 19.03, p = 0.002), *single support time* (χ^2^(5) = 14.14, p = 0.014) and *second double support time* (χ^2^(5) = 16.77, p = 0.005), but no significant effect was detected for *normalized walking speed* (χ^2^(5) = 8.88, p = 0.113) nor *whole stance time* (χ^2^(5) = 8.99, p = 0.109).

These results indicate that sagittal patterns are associated with spatio-temporal parameters. Mildly altered patterns (I and II) and Pattern VI, which show major kinematic abnormalities, preserve their spatio-temporal performance.

### Random forests differentiate “close-to-normal” gait cycles in HSP from healthy ones

**Fig 6:**
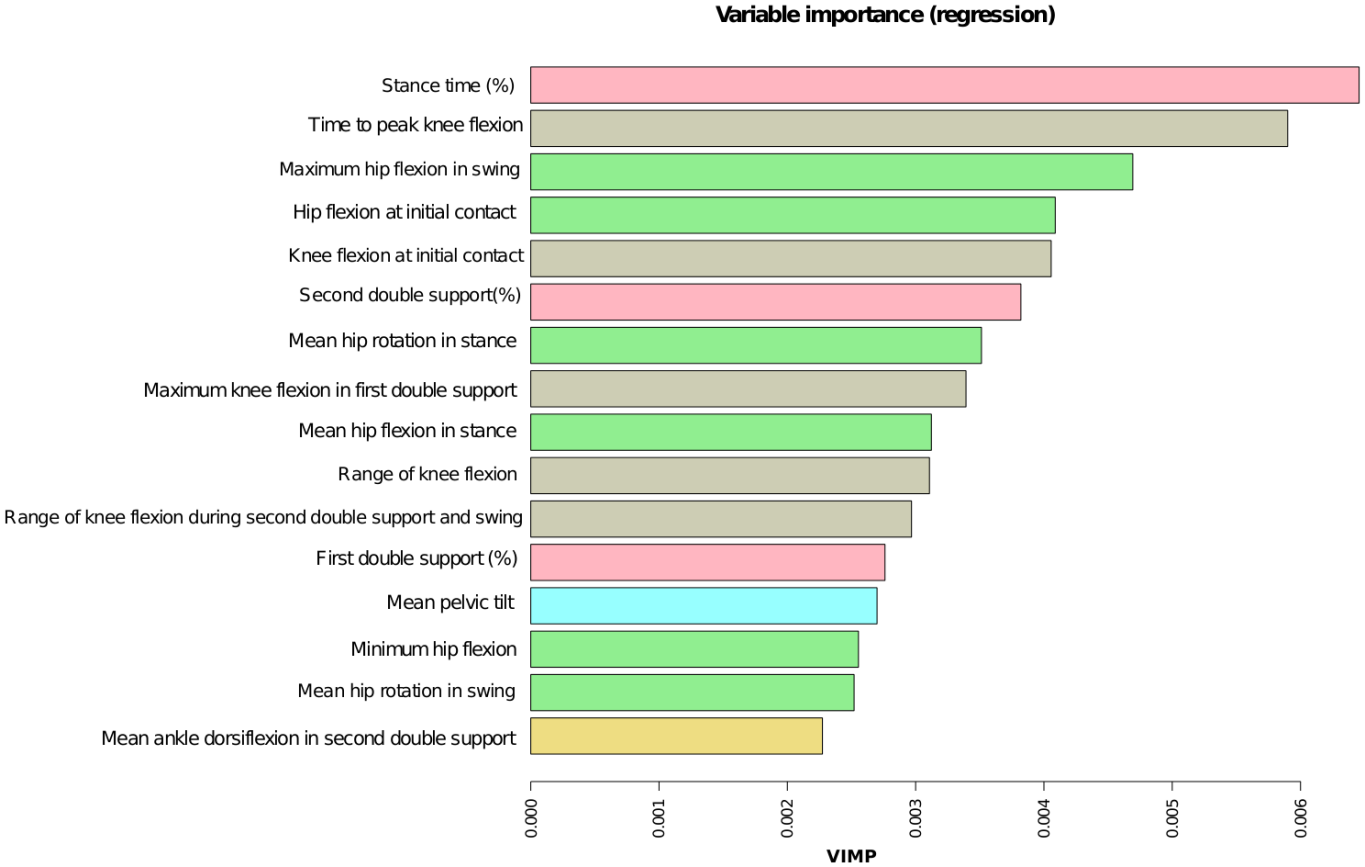
Importance of gait parameters for the random forest model to distinguish between cycles from HSP sagittal Pattern I and healthy controls. It is measured by Breiman-Cutler permutation variable importance (VIMP). *Stance time* and *time to peak knee flexion in stance* are the most important parameters to distinguish patients with sagittal Pattern I from healthy controls.

Although Pattern I cycles (red) show no clear differences from normalcy, random forests are able to discriminate between them. The best fitting model was the one with one variable at each split (area under the curve 0.979). The most important gait parameters to make this distinction are *stance time %*, *time to peak knee flexion*, *maximum hip flexion in swing*, *hip flexion in initial contact*, *knee flexion at initial contact and percentage of time of the second double support* (Fig 6).

**Fig 7:**
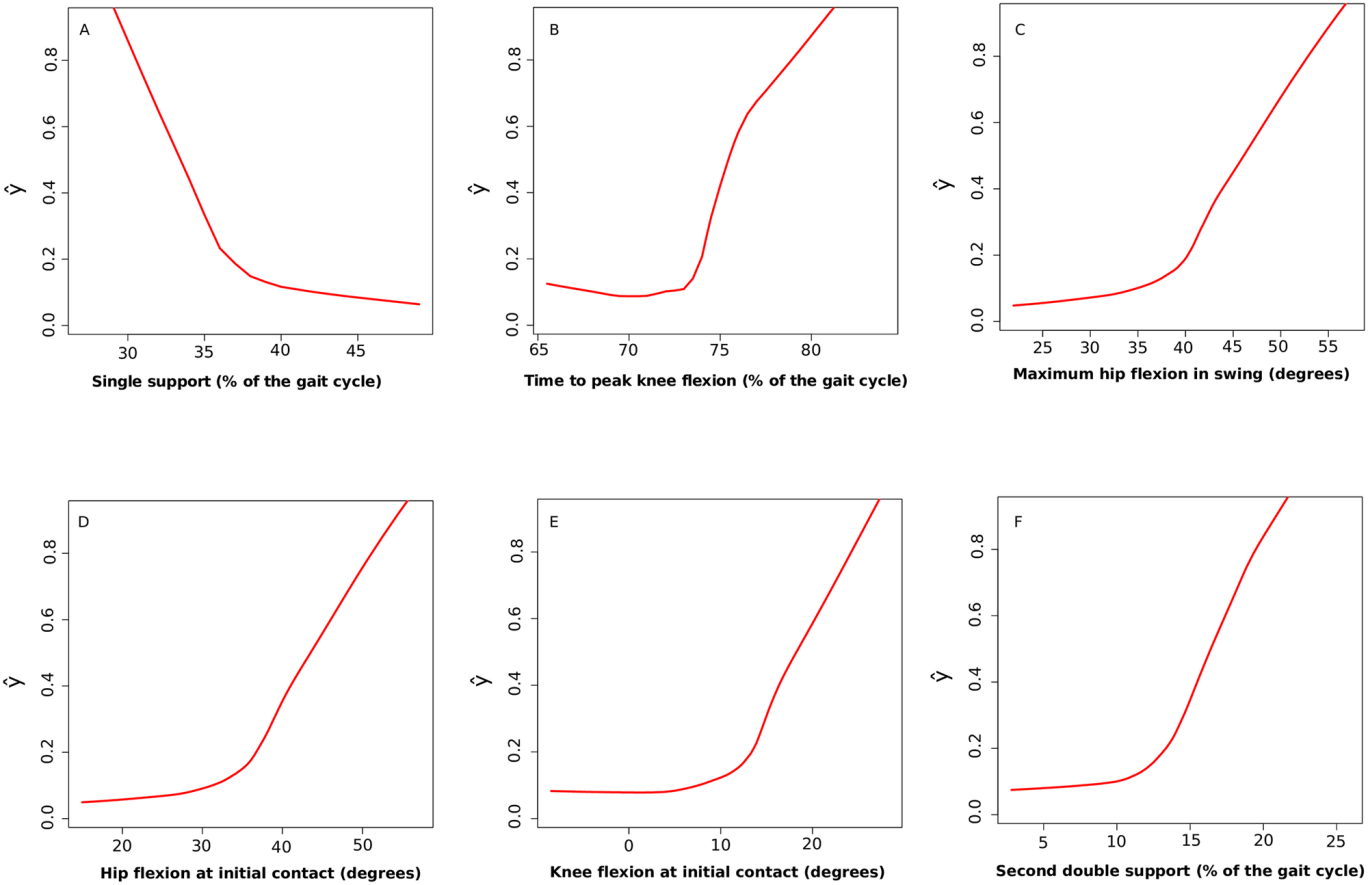
Relationships between model estimator ŷ (vertical axis) with values of the stance time (A), time to peak knee flexion (B), maximum hip flexion in swing (C), hip flexion at initial contact (D), knee flexion at initial contact (E) and second double support (E). Estimator ŷ is represented in the vertical axis, its range is from 0 to 1, with 0 representing minimum chance of being red and 1, maximum. On the horizontal axis, the value for the gait parameters. A) Longer percentage of single support time indicates a higher chance of being classified in the red pattern (values higher than 70% are indicative of disease); B) Percentage values of time to peak knee flexion greater than 75% of the cycle indicates the presence of HSP. C) High values of *maximum hip flexion in swing* (more than 45 degrees) indicate high possibility of presenting a mild form of HSP. D) The possibility of having HSP increases markedly when the values of *hip flexion at initial contact* are higher than 40 degrees. E) Values of knee flexion at initial contact higher than 20 degrees indicate high possibility of HSP presence. F) High percentage values in *double support* and *hip flexion at initial contact* indicate a greater chance of being affected.

Fig 7(A)–7(F) indicates the relationships between the values of those six parameters with a higher degree of importance for classification of cycles, with the model estimation (ŷ), that is an indicator of the possibility of belonging to the patients’ group. None of the parameters established linear relationships with model estimation. Cycles classified in Pattern I have longer duration of the stance and double support, more delayed *peak value of knee flexion*, higher value of *knee flexion at initial contact* and *larger hip flexion at initial contact and in swing* than healthy cycles.

### Sagittal patterns are related to severity of disease, disease duration and results of complementary tests in HSP

**Fig 8:**
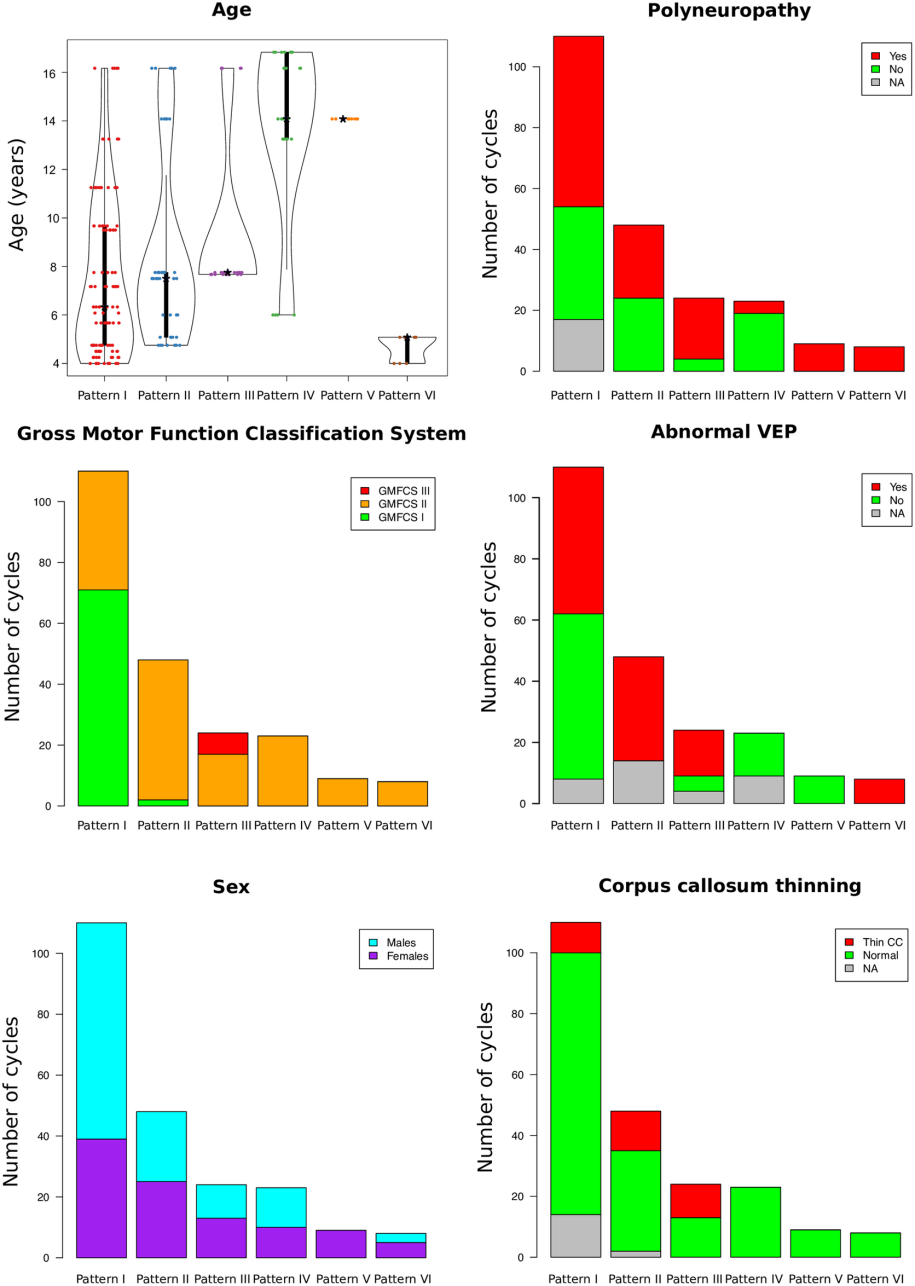
Distribution of clinical features between gait cycles for each gait pattern. Distribution of age between patterns were shown by means of violin plots. Frequencies of GMFCS, sex, polyneuropathy, abnormal VEP (visual evoked potentials) and corpus callosum thinning were shown by means of bar plots. See text for details.

The patterns in which gait cycles are classified differ by patients’ age, patients’ sex, patients’ GMFCS, presence of polyneuropathy, VEP abnormalities and by the presence of thin corpus callosum at brain MRI (see S3 Table and Figs 3 and 8). Cycles classified in Pattern I (in red) were used as reference in this part of the study. Interestingly, most of the cycles belonging to patients with mild impairment (GMFCS level I, children that are fully independent for displacement) are classified in this pattern, but some gait cycles matching patients with GMFCS level II are also classified in Pattern I. The other analysed clinical features are not related to Pattern I. Cycles classified in Pattern II (in blue) belong to patients with similar age and similar sex distribution as those of Pattern I. Most of the cycles classified in Pattern II match patients with GMFCS II (difficulties over long distances or other particular situations). Moreover, cycles in Pattern II tend to correspond more likely with patients showing a thin corpus callosum at MRI (OR = 3.39, p = 0.009). Cycles of Pattern III belong to older patients (age range: 6–16.8, p < 0.001) with a similar sex distribution, GMFCS II or III (but non-significant differences from Pattern I), with higher frequency of polyneuropathy (OR = 3.3, p = 0.042), of abnormalities in VEP (OR = 3.37, p = 0.028) and of corpus callosum thinning (OR = 7.22, p < 0.001) than those which use Pattern I. Cycles classified in Pattern IV correspond to older patients (age range 7.7–16.2, p = 0.027). They also belong to patients with lower frequency of polyneuropathy (OR = 0.139, p< 0.001). Pattern V groups together cycles from a single patient with an age of 14 years old, older than the average age of cycles from Pattern I. Cycles from Patterns VI correspond to younger patients than those who use Pattern I (age range: 4–5.1 years, p = 0.023).

### Random forests reveal relevant gait parameters in the relationships between gait impairment and clinical features

To understand better how the clinical features influence gait performance, we tested the capacity of random forest using the gait parameters as input to predict the age and the patients’ sex, GMFCS, presence or absence of polyneuropathy, presence of VEP abnormalities and the presence of thin corpus callosum at brain MRI (see Table 3). Based on gait parameters, random forests were able to differentiate patients with GMFCS I from patients with GMFCS II or III with significant accuracy (AUC = 0.987). In addition, random forests predicted age (Spearman’srho = 0.635) and the presence or absence of polyneuropathy (AUC = 0.721) and of thin corpus callosum at MRI (AUC = 0.763) with moderate accuracy. The predictions of patient’s sex and abnormal VEP by means of random forest seem to be as accurate as random selection.

**Table 3:** Goodness of fit of random forests used for the prediction of clinical features based on gait parameters.

Clinical feature	Mtry of the best model	Goodness of fit
Age	14	Spearman’s rho = 0.635
Sex	7	AUC = 0.527
Polyneuropathy	7	AUC = 0.721
GMFCS	14	AUC = 0.987
Abnormal VEP	7	AUC = 0.504
Thin corpus callosum at MRI	1	AUC = 0.763

**Fig 9:**
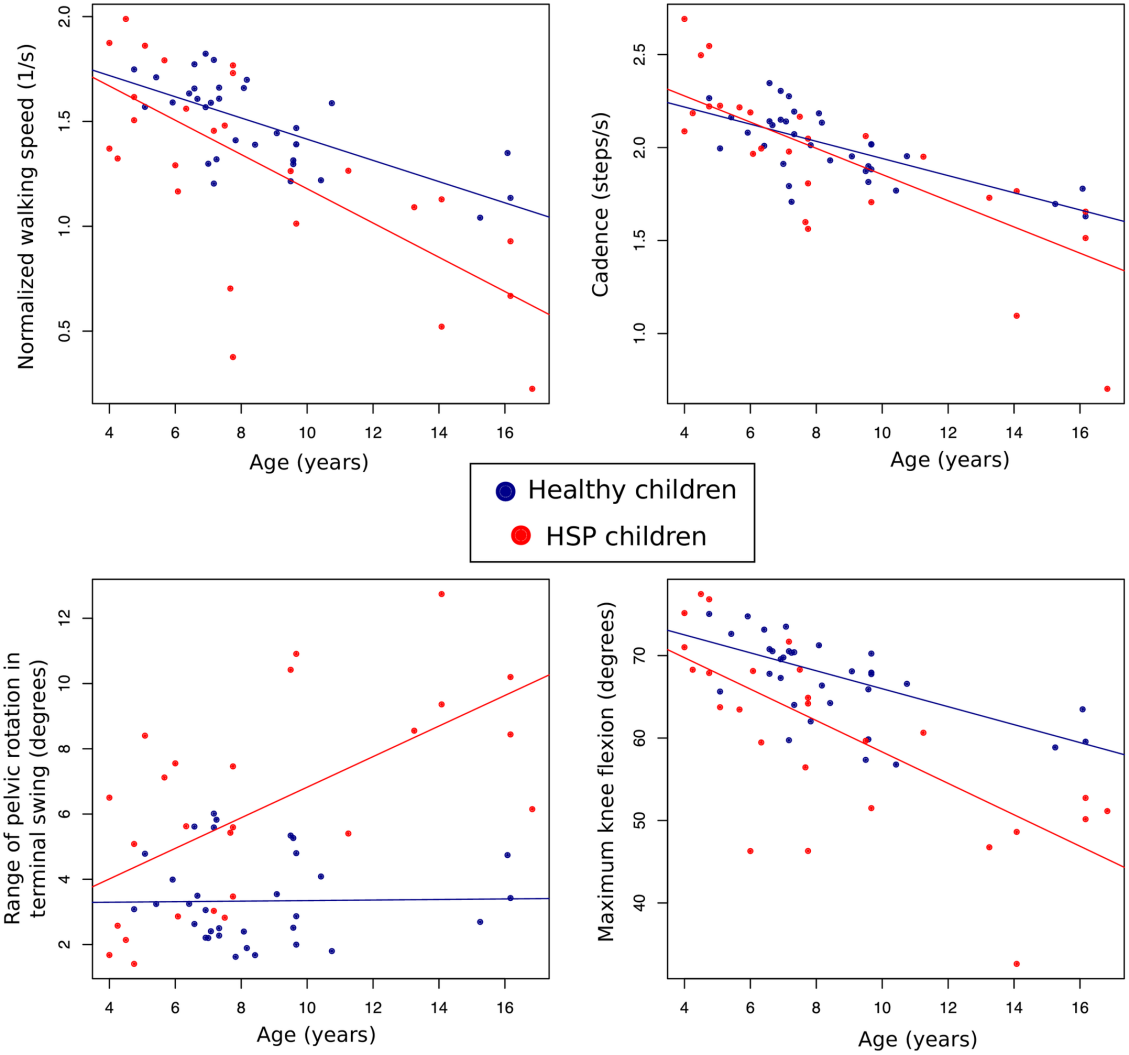
Relationships of the four most important gait parameters to predict age in HSP according to random forest model. Age is shown in the x-axis and each gait parameter in the y-axis. We represent healthy and HSP children data (blue and red points) and the adjusted linear mixed model for each group (blue and red lines). In the case of cadence (upper right), age and cadence are similarly related in both groups. In the case of normalized walking speed (upper left), the decrease of normalized walking speed with age tends to be higher in children with HSP, although it is not statistically significant (see text). Range of pelvic rotation in terminal swing (lower left) increases only with age in children with HSP, while it seems to remain stable in healthy subjects along the age spectrum. Maximum knee flexion (lower right) decreases with age both in HSP children and healthy children, but in the case of disabled children, it seems to decrease significantly faster.

The most important parameters for the prediction of age based on random forest model were normalized walking speed, cadence, range of pelvic rotation in terminal swing and maximum knee flexion (S4 Fig). In order to contrast whether the relationship between age and each parameter is significant, we adjusted robust linear regressions for the prediction of each gait parameter based on age, condition and the interaction of age and condition. *Normalized walking speed* and cadence decreases with increasing age (Fig 9). The effect of age on normalized walking speed is significant (normalized walking speed decreases 0.05 s^-1^ per year on average, 95% CI: 0.058 to 0.034, p < 0.001), but the difference in the effect of age between healthy and HSP children does not reach statistical significance (*normalized walking speed* decreases 0.03 s^-1^ per year more in HSP children than in healthy ones, on average, 95% CI: -0.013 to 0.039, p = 0.108). *Cadence* also diminishes with increasing age (cadence decreases 0.046 steps per second per year, 95% CI 0.03 to 0.058, p < 0.001), but at a similar pace in HSP and healthy children (p = 0.323). *Range of pelvic rotation in terminal swing* does not change with age in healthy children (p = 0.962), but significantly increases in HSP children (*range of pelvic rotation in terminal swing* increases by 0.471 degrees per year more in HSP than in healthy children, 95% CI 0.157 to 0.787, p < 0.001). On average, *maximum knee flexion* decreases with age in healthy children by 1.092 degrees per year (95% CI 0.673 to 1.511, p < 0.001), but it decreases 0.716 degrees per year more in HSP than in healthy subjects on average (95% CI 0.022 to 1.411, p = 0.043).

**Fig 10:**
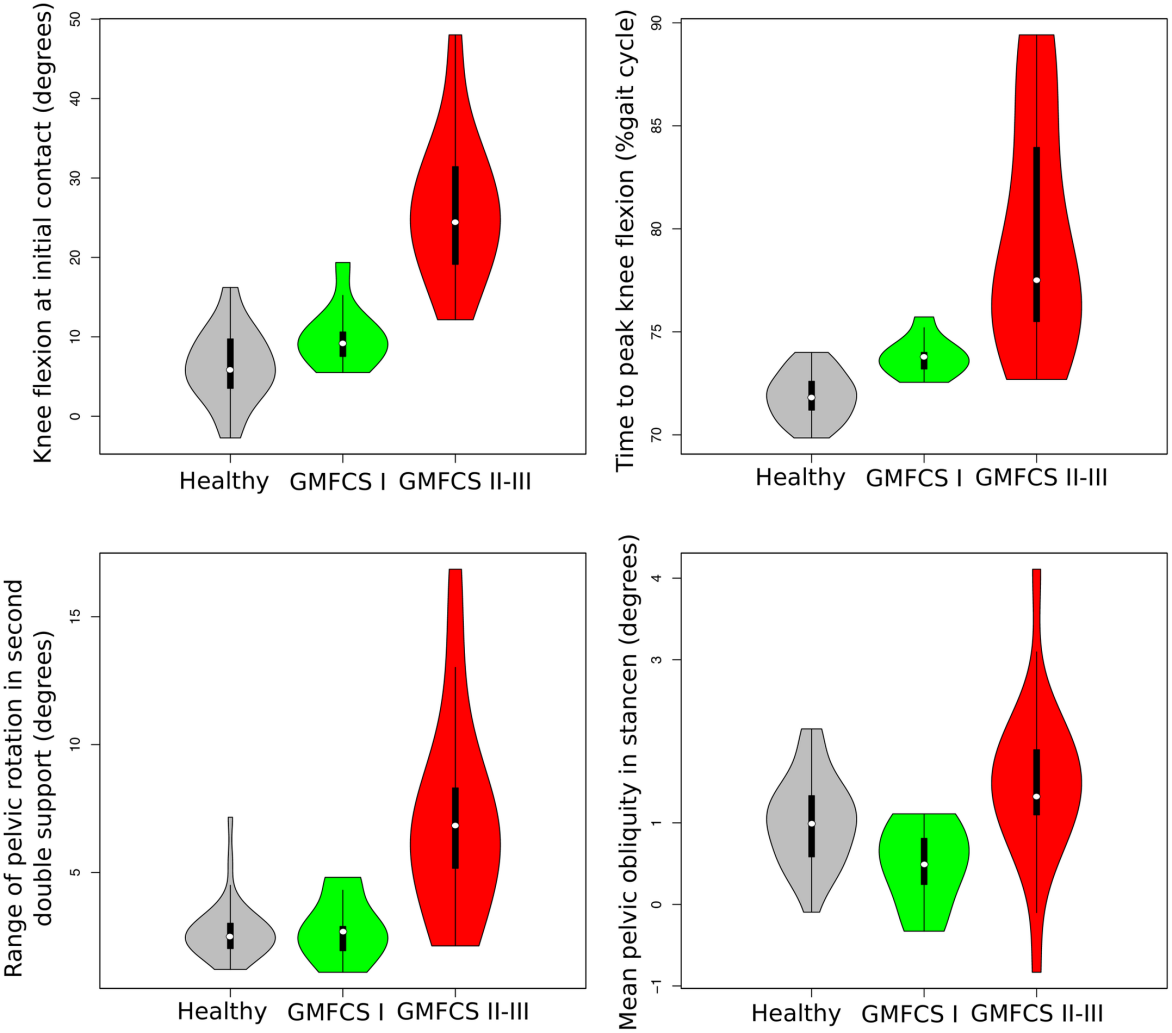
Violin plots of the four most important gait parameters to distinguish between patients with GMFCS I and GMFCS II-III according to random forest model. The vertical axis represents the value of the gait parameters studied. The shape of the violin plot depends on the distribution of the values of the gait parameters in each group. Grey violin plots show data distribution from healthy children, the green ones data distribution from children with HSP and GMFCS I and the red ones distribution from children with HSP and GMFCS II-III. In each violin plot, the white point represents the median value of each group; the vertical black line represents the range.

The most important parameters in the differentiation of patients with GMFCS I from those with GMFCS II-III were *knee flexion at initial contact*, *time to peak knee flexion*, *range of pelvic rotation in second double support* and *mean pelvic obliquity in stance* (see Fig 10). Patients with GMFCS I walk with a *knee flexion at initial contact* similar to healthy children, while those with more disability show increased angles of knee flexion at initial contact (see Fig 10)–mean difference GMFCS I versus GMFCS II: -10.2 degrees (-2.01 healthy standard deviations—SD), 95% CI: -18.3 to -3.2 degrees (-3.23 to -0.53 healthy SD). Patients with GMFCS I reach peak knee flexion at times which are equivalent to those of healthy children, while a proportion of patients with GMFCS II show increased time to peak knee flexion—mean difference GMFCS I versus GMFCS II: 5.94% of gait cycle (5.17 healthy SD), 95% CI: 4.11 to 8.58% of gait cycle (3.44 to 8.61 healthy SD). In addition, some patients with GMFCS II-III will also show *increased range of pelvic rotation in second double support*–mean difference GMFCS I versus GMFCS II: 4 degrees (2.95 healthy SD), 95% CI: 2.5 to 5.7 degrees (1.75 to 4.37 healthy SD)–and increased *mean pelvic obliquity in stance*–mean difference GMFCS I versus GMFCS II: 0.99 degrees (1.82 healthy SD), 95% CI: 0.6 to 3% of gait cycle (0.36 to 1.68 healthy SD)–, indicating a higher implication of pelvic movements in gait transfer.

**Fig 11:**
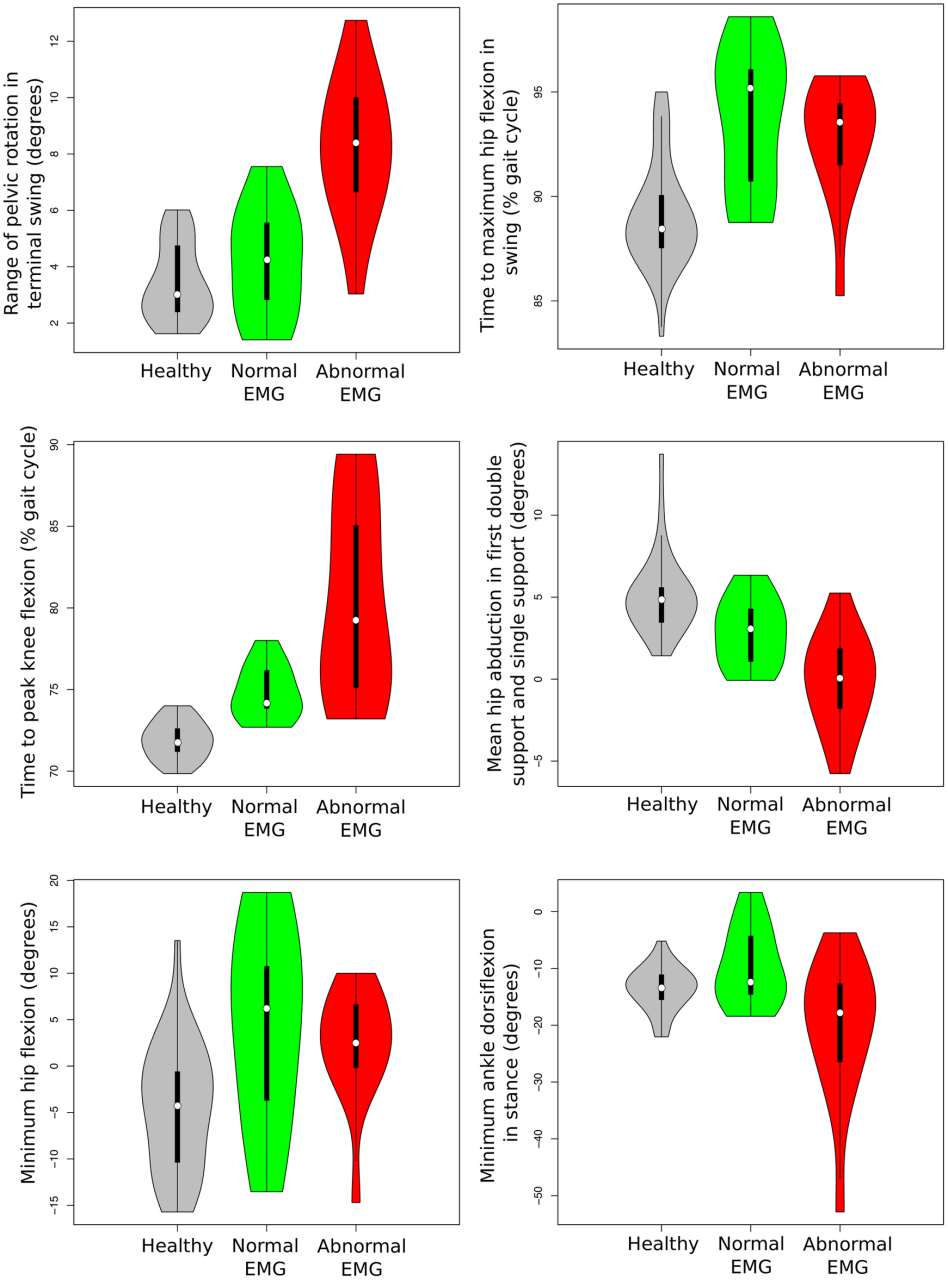
Violin plots of the six most important gait parameters to distinguish between patients with normal and abnormal EMG. The vertical axis represents the value of the gait parameters studied. The shape of the violin plot depends on the distribution of the values of the gait parameters in each group. Grey violin plots show data distribution from healthy children, the green ones data distribution from children with HSP and normal EMG and the red ones distribution from children with HSP and abnormal EMG. In each violin plot, the white point represents the median value of each group; the vertical black line represents the range.

Polyneuropathy was related to the *range of pelvic rotation in terminal swing*, *time to maximum hip flexion in swing*, *time to peak knee flexion*, *mean hip abduction in first double support and single support*, *minimum hip flexion and minimum ankle dorsiflexion in stance* (see Fig 11). Patients with polyneuropathy had increased *range of pelvic rotation in terminal swing*–mean difference patients with versus without polyneuropathy: 4.04 degrees (2.98 healthy SD), 95% CI: 2.45 to 5.65 degrees (1.84 to 4.23 healthy SD)–and *increased time to peak knee flexion*–mean difference patients with versus without polyneuropathy: 5.53% of gait cycle (4.82 healthy SD), 95% CI: 2.32 to 12.15% of gait cycle (2.77 to 9.94 healthy SD). In contrast to patients with polyneuropathy (see Fig 11), some patients with normal EMG reach values of *minimum hip flexion* and *time to peak hip flexion in swing* higher than healthy subjects, but there are no differences between the mean values of patients with and without polyneuropathy in these two parameters (*minimum hip flexion*: mean difference -0.34 degrees 95% CI -1.5 to 0.74 degrees; *time to maximum hip flexion in swing*: mean difference: -0.37 degrees 95%CI -1.99 to 0.42 degrees). *Mean hip abduction in first double support and single support* was significantly lower in patients with polyneuropathy—mean difference patients with versus without polyneuropathy: -2.98 degrees (-1.24 healthy SD), 95% CI: -5.40 to -1.40 degrees (-2.07 to -0.6 healthy SD). *Minimum ankle dorsiflexion in stance* was also lower in those patients with abnormal EMG—mean difference patients with versus without polyneuropathy: -10.8 degrees (-2.8 healthy SD), 95% CI: -20.43 to -4.5 degrees (-5.8 to -1.3 healthy SD).

**Fig 12:**
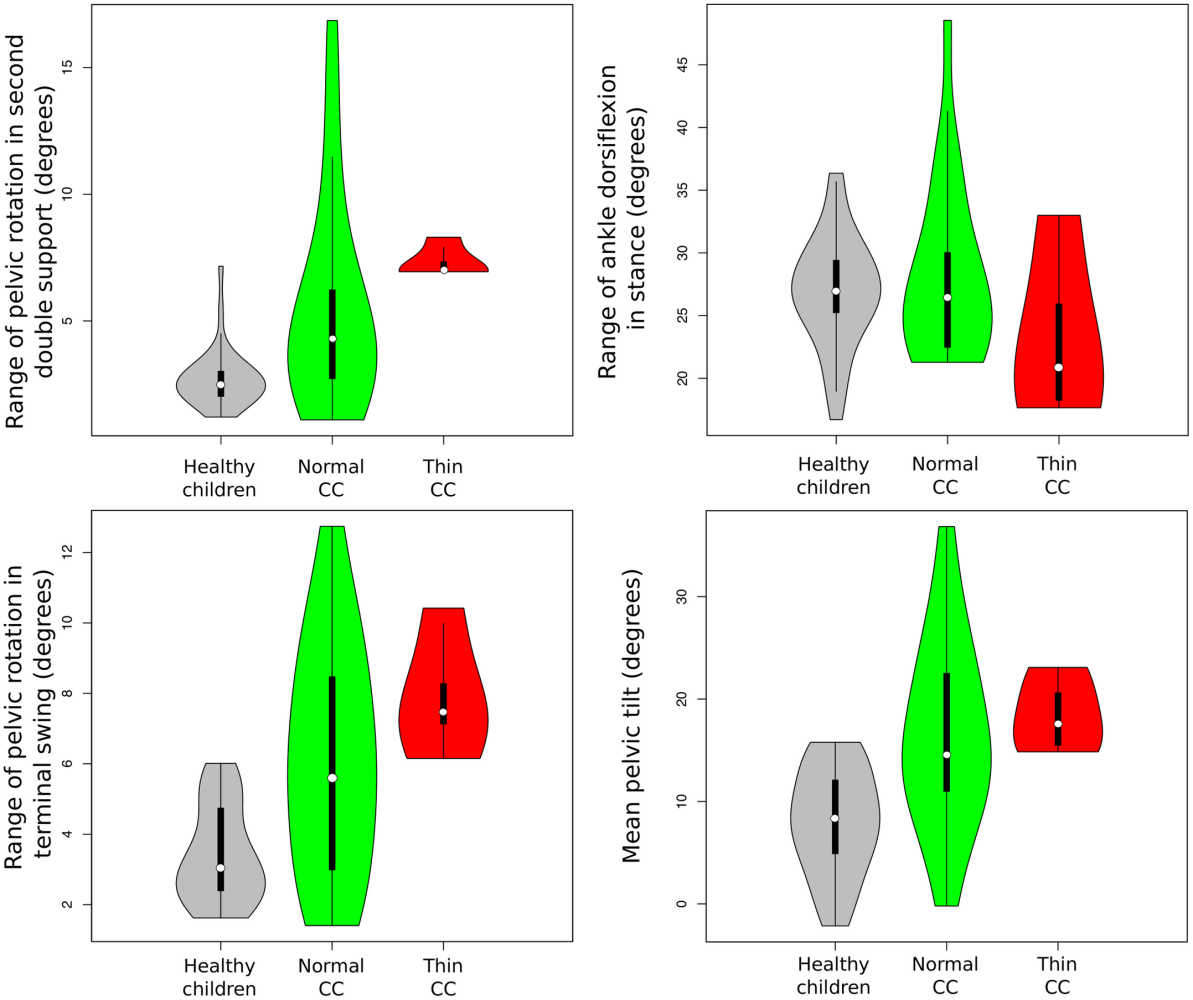
Violin plots of the four most important gait parameters to distinguish between patients with apparently normal corpus callosum and patients with thin corpus callosum according to random forest model. The vertical axis represents the value of the gait parameters studied. The shape of the violin plot depends on the distribution of the values of the gait parameters in each group. Grey violin plots show data distribution from healthy children, the green ones data distribution from children with HSP and apparently normal corpus callosum and the red ones distribution from children with HSP and thin corpus callosum. In each violin plot, the white point represents the median value of each group; the vertical black line represents the range.

The parameters that were important in the prediction of the presence of thin corpus callosum (see Fig 12) were: *range of pelvic rotation in second double support*, *range of ankle dorsiflexion in stance*, *range of pelvic rotation in terminal swing and mean pelvic tilt*. None of the differences of means in these parameters between patients with apparently normal corpus callosum and thin corpus callosum were statistically significant, but differences do exist in the distribution of these parameters between these two groups. All HSP children (see Fig 12) with thin corpus callosum have increased *ranges of pelvic rotation in second double support* and *in terminal swing*, in contrast to patients with apparently normal corpus callosum, who can walk with increased ranges of pelvic rotation but also with values in the range of those shown by healthy children. *Mean pelvic tilt* in all HSP children with thin corpus callosum is increased in comparison to healthy children, but HSP children with apparently normal corpus callosum may show increased or normal values. The *range of ankle dorsiflexion in stance* in HSP children with thin corpus callosum is usually lower than healthy average but in patients with apparently normal corpus callosum, this value is variable.

## Discussion

We have developed a new approach to determine and to understand gait patterns in HSP, which is a rare neurological disease. We have used a parameter-free classification approach for the whole gait cycle. We have used random forests to relate this classification to interpretable gait parameters that are widely used in clinical studies. This approach has revealed six main patterns in a sample of HSP children and has detected the important gait parameters that distinguish them. In addition, we have used random forests to discriminate between cycles from healthy children and those cycles from HSP children with milder gait abnormalities, and detected gait alterations while the disease remains with minimal expression. We have also related the above-mentioned gait patterns to age (as an indicator of disease duration), sex, GMFCS stage (as indicator of disease severity), presence of polyneuropathy, abnormal VEP and presence of thin corpus callosum. Finally, we have analyzed the effect of those clinical features on gait performance revealing specific gait abnormalities linked to each clinical item.

The existence of gait patterns in a disease indicates different types of impairment and different biomechanical alterations that can represent different therapeutic targets. Our description of patterns and the use of random forests to detect which gait parameters are most discriminating across patterns helps us to surmise which biomechanical alterations specifically underlie featured kinematic abnormalities of each pattern. Pattern I is very similar to healthy gait and its differences require further discussion after describing the rest. The kinematic abnormalities observed in cycles from Pattern II (in blue) may indicate the presence of iliopsoas muscle spasticity (anterior pelvic tilt and increased hip flexion) and spasticity or overactivity of the hamstrings (increase of knee flexion at initial contact). Some patients also show decreased values of maximum knee flexion which may be secondary to overactivity of the hamstrings or to abnormal quadriceps function[10]. In spite of these kinematic abnormalities in Pattern II, spatio-temporal parameters are relatively spared. Pattern III (in purple) is mainly characterized by a severe alteration in knee flexion. Quadriceps spasticity that could be accompanied by spasticity of the triceps surae muscle or a proprioceptive deficit may lead to knee *recurvatum* and decreased and delayed peak value of knee flexion. Pattern IV (in green) resembles crouch gait, which arises from poorly understood mechanisms. According to studies in CP, crouch gait may be due to weakness of hip extensors (gluteus medius and maximum), vastii and soleus muscles [27, 28]. Gait changes in Pattern V may arise from spasticity of iliopsoas and other hip flexor muscles (which may lead to anterior pelvic tilt and decreased hip extension) and spasticity of triceps surae muscles (that may induce knee *recurvatum* and *equinus* foot), probably in combination with impaired quadriceps function. Pattern VI (brown colour) is similar to so-called “jump knee” pattern, previously reported in CP patients[29]. The findings are suggestive of iliopsoas muscle spasticity with overactive hamstrings and rectus femoris muscles and spasticity of the triceps surae muscles [30, 31].

Gait Pattern I is very similar to the gait of healthy children. Cycles show normal values of *walking speed*, of *cadence* and of most kinematic parameters. We had to use a regressive random forest to adequately differentiate cycles from Pattern I and those from healthy children. Beyond this, the random forest allows better characterization of the “closer-to-normal” pattern, distinguishing gait parameters that indicate the specific biomechanical mechanisms underlying mild forms of HSP. Mildly affected HSP patients are characterized by abnormal gait cycle time distributions of stance (increased *stance time* with longer *double supports* could be the consequence of instability, probably as a combination of proprioceptive impairment, loss of strength and selective control alteration), delayed *peak knee flexion* and higher value of *knee flexion at initial contact* (probably related to mild gastrocnemius spasticity and/or gait cycle distribution alterations) and increased *hip flexion at initial contact* and maximum *hip flexion in swing* (that could be related to a slight spasticity in iliopsoas or may be an attempt to compensate for decreased advancement of the limb[10, 32]. However, these abnormalities are generally mild, do not occur in every mildly affected patient and their description only highlights the importance of further studies to analyse the causes of these abnormalities in the early phase of HSP.

Some patients use different patterns for the contralateral limb or even different patterns in the same limb. Gait asymmetry has not previously been reported, but this finding is perhaps to be expected due to the classically described asymmetry in spasticity in HSP patients[33]. The use of different patterns by the same limb in a patient may be secondary to the capacity to use several kinematic strategies, or simply to the fact that the cycles from these patients are borderline in the classification between groups.

Although there is a general trend to show impairment in gait distribution (longer stance time and double support times compared to those of healthy individuals), the capacity of HSP patients to maintain walking speed, cadence and gait cycle distribution near to those of the normal group differs between the patterns. Pattern IV (green), V (orange) and part of Pattern III (purple) show major impairment of spatio-temporal parameter values (low *walking speed* and *cadence* and longer *double support*); however, it is noteworthy that Patterns I and II, which are close to normal in terms of kinematics, and Pattern VI (brown, only right-side cycles), which show major alterations in flexion behaviour of the different joints during gait, show spatio-temporal parameters near to normal values. To our knowledge, the effectiveness of gait patterns to maintain spatio-temporal parameters had not been previously studied and indicates the complexity of the relationship between kinematic parameters and spatio-temporal parameters in HSP, where spatio-temporal degree of alteration could be the consequence of adaptation to instability (probably as a combination of proprioceptive impairment, loss of strength and selective control alteration)[8], or to kinematic impairment. We also hypothesize that causal relationships between spatio-temporal and kinematic parameters are bidirectional, so that some kinematic changes may also be produced by compensatory abnormal cycle distribution or decreased walking speed.

Wolf *et al*. [14] had previously described the presence of five flexion patterns in a cohort of adults and children with HSP. The patterns they found are similar to ours (I–“close to normal”, III–“stiff knee”, IV–“crouch gait”, V–“*recurvatum* knee” and VI–“jump knee”) with the exception of Pattern II, which was not found by Wolf *et al*. Our study and that of Wolf *et al*[14] both highlight the interest in looking for order in the heterogeneous gait disorder of HSP, but significant differences in aims, methods and design make them complementary. In our study, we only included children at lower stages of GMFCS, and in consequence, the distribution of gait patterns within the samples diverges. Wolf *et al*.[14] found that the pattern similar to normal gait was uncommon, but in our series, the two most common patterns (I and II) were the closest to normalcy. From the data analysis point of view, we used a different approach with DTW algorithm, which avoids assumptions such as linearity in the relationships and similar temporal distribution between cycles, and with hierarchical clustering, which produces a sequence of clustering without pre-supposing a previous number of clusters. Moreover, as our aims are different to those of Wolf *et al*.[14], we applied random forests to understand the differences in gait parameters across patterns.

A further novelty of our study is the association of the gait patterns with some clinical features that frequently concur in HSP. Cycles classified in Pattern I mostly correspond to the mildest affected patients at GMFCS I stage, suggesting that the preservation of gait performance is critical for the maintenance of functionality. Pattern II mainly distinguishes cycles from patients at GMFCS II stage. The rest of the patterns correspond to cycles from patients at GMFCS II or III. Age, as a proxy of disease duration, is a good predictor of the gait pattern. Patterns I and II include mainly cycles from younger patients (although some from patients over the whole age spectrum of our sample are included as well). Hence, those Patterns might be used at early phases of the disease while patterns III, IV and V would be used at more advanced phases across the disease progression. Some adolescent patients may use Pattern I or II. Should these results be confirmed after longitudinal studies that addressed long-term gait changes in pediatric HSP, changes between patterns could be used as indicators of the rate of disease progression. Polyneuropathy was most common in those patients classified in Patterns III, I and II. In this context, gait parameters are better predictors for polyneuropathy than gait patterns. VEP abnormalities were significantly more frequent in patients classified in Pattern III (severe knee flexion abnormalities) and although not significant, they were absent in Patterns IV and V. The reason why this occurs is unclear but we suggest that VEP abnormalities may occurs differential neurodegenerative phenomena that would impair motor control in a differential way.

Random forests showed that GMFCS stages are closely related to gait performance, in particular with knee flexion and non-sagittal pelvic movements. The most severely affected patients (GMFCS II and III) show delay of peak knee flexion with increased *knee flexion at initial contact*, suggesting an inadequate hamstring activity during swing or hamstring spasticity[10]. These may represent important indicators of increasing HSP disability. This is congruent with our findings described above in the sense that normal knee flexion at initial contact seemed to be a feature that was important to distinguish Pattern I, used mainly by patients at GMFCS I stages. Increased pelvic rotation and pelvic obliquity are probably reflect compensatory mechanisms that patients at GMFCS II and III recruit to maintain translation in spite of their lower limbs weakness and impaired motor control [10]. These gait parameters could be useful to monitor gait impairment in paediatric HSP. The importance of knee flexion and pelvic non-sagittal movements in the progression of HSP is also confirmed by the differences that we found in the relationship of the range of pelvic tilt at terminal swing and maximum knee flexion with age, when comparing HSP and healthy children.

Random forests were able to detect gait differences between patients with and without polyneuropathy and with and without corpus callosum thinning. We believe that the impact on gait of peripheral nerve disease has been overlooked in HSP, probably because in most of the cases it is mild and subclinical. However, we have demonstrated that its presence could modify (or at least, be related to) gait performance. Our findings are consistent with the gait alterations found in Charcot-Marie-Tooth disease[34]. With regard to the relationship between gait parameters and corpus callous thinning, we interpret it with caution. Probably this relationship arises as a confounding effect, since it could be linked to genetic forms that have that condition, without there being a direct causal relationship between gait and corpus callosum thinning.

One of the limitations of our study is the still low yield of genetic studies in the context of the high number of genes described as being involved in these rare disorders[35]. It is too difficult to recruit groups of patients with mutations in the same or for specific gene mutations. These facts limited the possibility of performing a sub-analysis to correlate specific gait changes with specific genetic types. However, these disorders share a common pathogenic mechanism[36], which provides a rationale for gait comparisons and classification even with this high genetic heterogeneity.

Our approach and findings on HSP may have an impact on the design of current therapeutic strategies (botulinum toxin, physical therapy, etc.). For example, botulinum toxin injections in hamstrings could be a rational treatment for patients showing Pattern II, IV or VI, in which hamstring overactivity/spasticity may play an important role. In this case, according to our results, the values of knee flexion at initial contact could represent a useful parameter to monitor response to this approach.

The clinical usefulness of the patterns should be tested in longitudinal studies which evaluate treatment. In addition, studies with long-term follow up are critical to fully demonstrate the findings of our study. In these studies, the progression of patients along different patterns could be evaluated and the role of the suggested gait parameters to monitor the progression of the disease could be confirmed.

The development of accurate methods for identifying gait patterns in a disorder is important because classifying patient performance in relevant categories may assist with clinical decision-making[37]. DTW distance calculation is a robust method that allows the quantification of differences in angular joint movement between cycles, and that has been widely used in other disciplines that deal with signal analysis. However, to really make patterns clinically useful, we need to provide specific parameters that distinguish the classification groups. This allows quick identification in clinical settings with observational gait analysis and reliable and automatic identification in research, and suggests plausible underlying mechanisms of specific gait alterations for each pattern. Sometimes, finding those parameters is difficult with classical statistical methods. We here propose the use of machine learning techniques such as random forests as a second step to reveal the most important parameters to explain why patterns exist when performing a parameter-free classification by DTW. Moreover, this novel approach could be easily transferred to the study of gait alterations in other diseases and may be particularly suitable for rare neurological disorders. From the data mining perspective, this approach is also an example of the usefulness of combining different data mining and machine learning techniques to address problems in the biomedical field.

In conclusion, we have demonstrated the presence of six different sagittal gait patterns in HSP paediatric patients by means of a combination of calculation of DTW distance and hierarchical clustering with random forests. We have defined those parameters that are important to distinguish gait patterns and we have also identified those gait parameters that are relevant to differentiate the mildest forms of the disease from the normal group. In addition, we have studied the effects of age (duration of disease), sex, severity of disease (GMFCS) and comorbidities on the onset of the different patterns, and we have identified specific gait parameters that can predict age, degree of disability and the presence of polyneuropathy.

## Supporting information

S1 Table Clinical features of HSP patients. (PDF)

S2 Table Distribution of sagittal patterns in left and right cycles of each patient. (PDF)

S3 Table Clinical characteristics of gait cycles according to gait pattern and results of general linear mixed models. (PDF)

S1 Fig Markers’ position. (TIF)

S2 Fig Chart flow of the methodological steps used in the present work. (PDF)

S3 Fig Violin plots of spatio-temporal parameters for each gait pattern detected. (Pattern I, in red; Pattern II, in blue; Pattern III, in purple; Pattern IV, in green; Pattern V, in orange; Pattern VI in brown.) and reference group (in grey). The graphs on the left show the results for left cycles; the graphs of the right show the results for right cycles. The vertical axis represents the value of the spatio-temporal parameters studied. The unit for each spatio-temporal parameter is summarized in Table 1. In each violin plot, the white point represents the average value of this pattern, the vertical black line represents the range. The shape of the violin plot depends on the distribution of the values of the spatio-temporal variable in the cycles classified in a particular pattern.
(TIF)

S4 Fig Importance of gait parameters for the random forest models to predict patients’ features. Importance is measured by Breiman-Cutler permutation variable importance (VIMP). Notice different scales in each random forest. Different colours indicate different joints (blue for pelvis, green for hip, brown for knee, and yellow for ankle parametes) and spatio-temporal parameters (pink).
(TIF)

S1 Data Gait data from HSP children. (ZIP)

S2 Data Gait data from healthy children. (ZIP)
