# Supplementary material for: Gait phenotypes in paediatric hereditary spastic paraplegia revealed by dynamic time warping analysis and random forests
Source: PLoS One. 2018 Mar 8;13(3):e0192345. doi: 10.1371/journal.pone.0192345 (PMC5843164; doi:10.1371/journal.pone.0192345)
Supplement: S1 Table — (PDF) [file pone.0192345.s001.pdf]

	Age (years)	Sex	Genetic information (gene or inheritance pattern)	Central motor conduction	Polyneuropathy signs in EMG	Epilepsy	Somatosensory evoked potentials	Auditory evoked potentials	GMFCS
P1	7	Male	AD	Altered	Incipient	No	Altered	Normal	2
P2	5	Male	AR/ de novo	Altered	No	No	Altered	Normal	2
P3	8	Male	SPG4	NA	No	NA	NA	NA	2
P4	10	Male	AR	Altered	Incipient	Yes	Altered	NA	2
P5	11	Male	SPG10	Altered	Mild axonal	No	Altered	Normal	1
P6	14	Female	SPG4	Altered	Mild axonal	No	Altered	NA	2
P7	6	Female	AD	Altered	No	No	Altered	Normal	1
P8	8	Male	AD	Altered	No	No	Normal	Normal	1
P9	5	Female	SPG3A	NA	Incipient	No	Altered	Normal	2
P10	6	Male	AR/Novo	Altered	No	No	Altered	Normal	1
P11	6	Male	AD	Altered	Mild axonal	No	Altered	Altered	1
P12	8	Male	AR	NA	Incipient	No	NA	NA	2
P13	17	Male	SPG11	Altered	Axonal	No	NA	NA	3
P14	8	Female	AR/ Mitochondrial	Altered	No	No	Altered	Normal	2
P15	6	Male	AR	Altered	No	No	NA	NA	2
P16	4	Male	AD	Altered	No	No	NA	Normal	1
P17	4	Female	AR/ de novo	Altered	NA	No	NA	NA	1
P18	5	Female	AR/ de novo	Altered	No	No	Normal	Normal	1
P19	5	Male	AR/ de novo	Altered	NA	No	NA	NA	1
P20	14	Female	SPG4	Altered	Axonal	No	NA	Normal	2
P21	16	Female	AR/ de novo	Altered	Axonal	No	NA	Normal	2
P22	13	Female	SPG4	Altered	Mild Axonal	No	NA	Normal	2
P23	10	Female	AR/ Mitochondrial	Altered	Incipient	No	Altered	Normal	2
P24	8	Female	SPG3A	Altered	No	No	Altered	Normal	2
P25	16	Male	AR/Novo	Altered	Mild axonal	No	Altered	Normal	2
P26	4	Male	AR/Novo	Altered	Doubtful	No	Altered	Normal	2
