# Supplementary material for: Gait phenotypes in paediatric hereditary spastic paraplegia revealed by dynamic time warping analysis and random forests
Source: PLoS One. 2018 Mar 8;13(3):e0192345. doi: 10.1371/journal.pone.0192345 (PMC5843164; doi:10.1371/journal.pone.0192345)
Supplement: S2 Table — (PDF) [file pone.0192345.s002.pdf]

<table>
<tr><th></th><th colspan="2">Left cycles</th><th colspan="2">Right cyles</th></tr>
<tr><td>P1</td><td colspan="2">1</td><td colspan="2">1</td></tr>
<tr><td>P2</td><td>1</td><td>2</td><td colspan="2">2</td></tr>
<tr><td>P3</td><td colspan="2">4</td><td colspan="2">4</td></tr>
<tr><td>P4</td><td colspan="2">1</td><td colspan="2">1</td></tr>
<tr><td>P5</td><td colspan="2">1</td><td colspan="2">1</td></tr>
<tr><td>P6</td><td colspan="2">3</td><td colspan="2">2</td></tr>
<tr><td>P7</td><td colspan="2">1</td><td colspan="2">1</td></tr>
<tr><td>P8</td><td>1</td><td>2</td><td>1</td><td>2</td></tr>
<tr><td>P9</td><td colspan="2">2</td><td colspan="2">6</td></tr>
<tr><td>P10</td><td colspan="2">1</td><td colspan="2">1</td></tr>
<tr><td>P11</td><td colspan="2">1</td><td colspan="2">1</td></tr>
<tr><td>P12</td><td colspan="2">2</td><td colspan="2">2</td></tr>
<tr><td>P13</td><td colspan="2">3</td><td colspan="2">3</td></tr>
<tr><td>P14</td><td colspan="2">4</td><td colspan="2">4</td></tr>
<tr><td>P15</td><td colspan="2">3</td><td colspan="2">2</td></tr>
<tr><td>P16</td><td colspan="2">1</td><td colspan="2">1</td></tr>
<tr><td>P17</td><td colspan="2">1</td><td colspan="2">1</td></tr>
<tr><td>P18</td><td colspan="2">1</td><td colspan="2">1</td></tr>
<tr><td>P19</td><td colspan="2">1</td><td colspan="2">1</td></tr>
<tr><td>P20</td><td colspan="2">5</td><td colspan="2">5</td></tr>
<tr><td>P21</td><td colspan="2">3</td><td colspan="2">2</td></tr>
<tr><td>P22</td><td colspan="2">3</td><td colspan="2">1</td></tr>
<tr><td>P23</td><td colspan="2">1</td><td colspan="2">1</td></tr>
<tr><td>P24</td><td colspan="2">2</td><td colspan="2">2</td></tr>
<tr><td>P25</td><td colspan="2">1</td><td colspan="2">4</td></tr>
<tr><td>P26</td><td colspan="2">Outlier</td><td colspan="2">6</td></tr>
</table>

Table S2. Distribution of sagital patterns in left and right cycles of each patient. Background colors correspond to the colour of the pattern used in figure 2.
