# Supplementary material for: Gait phenotypes in paediatric hereditary spastic paraplegia revealed by dynamic time warping analysis and random forests
Source: PLoS One. 2018 Mar 8;13(3):e0192345. doi: 10.1371/journal.pone.0192345 (PMC5843164; doi:10.1371/journal.pone.0192345)
Supplement: S3 Table — (PDF) [file pone.0192345.s003.pdf]

	**Pattern 1**	**Pattern 2**	**Pattern 3**	**Pattern 4**	**Pattern 5**	**Pattern 6**	**Total**
Number of cycles	110	48	24	23	9	8	223
Number of patients with at least one cycle with this pattern	15	8	5	3	1	2	---
**Age**<br>Median (range)<br>*p-value*	6.3 (4-16.2)<br>*Reference*	7.5 (4.8-16.2)<br>*NS*	14.1 (6-16.8)<br>*<0.001*	7.8 (7.7-16.2)<br>*0.027*	14.1 (14.1)<br>*<0.001*	5.1 (4-5.1)<br>*0.023*	7.6 (4-16.9)<br>*<0.001*
**Sex**<br>F/M<br>*p-value*	39/71<br>*Reference*	25/23<br>*NS*	13/11<br>*NS*	10/13<br>*NS*	9/0<br>*NS*	5/3<br>*NS*	101/122<br>*<0.001*
**GMFCS**<br>I/II/III<br>*p-value*	71/39/0<br>*Reference*	2/46/0<br>*<0.001*	0/17/7<br>*NS*	0/23/0<br>*NS*	0/9/0<br>*NS*	0/8/0<br>*NS*	73/143/7<br>*<0.001*
**Polyneuropathy**<br>Yes/No/NA<br>*p-value*	56/37/17<br>*Reference*	24/24/0<br>*NS*	20/4/0<br>*0.042*	4/19/0<br>*<0.001*	9/0/0<br>*NS*	8/0/0<br>*NS*	122/84/17<br>*<0.001*
**Altered VEP**<br>Yes/No/NA<br>*p-value*	48/54/8<br>*Reference*	34/0/14<br>*NS*	15/5/4<br>*0.028*	0/14/9<br>*NS*	0/9/0<br>*NS*	8/0/0<br>*NS*	106/82/35<br>*<0.001*
**Thin corpus callosum**<br>Yes/No/NA<br>*p-value*	10/86/14<br>*Reference*	13/33/2<br>*0.009*	11/13/0<br>*<0.001*	0/23/0<br>*NS*	0/9/0<br>*NS*	0/8/0<br>*NS*	34/173/16<br>*<0.001*

F: female; GMFCS: Gross Motor Function Classification System; M: male; NS: non-significant comparison; VEP: visual evoked potential.

Table S3: Clinical characteristics of gait cycles according to gait pattern and results of general linear mixed models.
