# Supplementary figures and images for: Gait phenotypes in paediatric hereditary spastic paraplegia revealed by dynamic time warping analysis and random forests
Source: PLoS One. 2018 Mar 8;13(3):e0192345. doi: 10.1371/journal.pone.0192345 (PMC5843164; doi:10.1371/journal.pone.0192345)

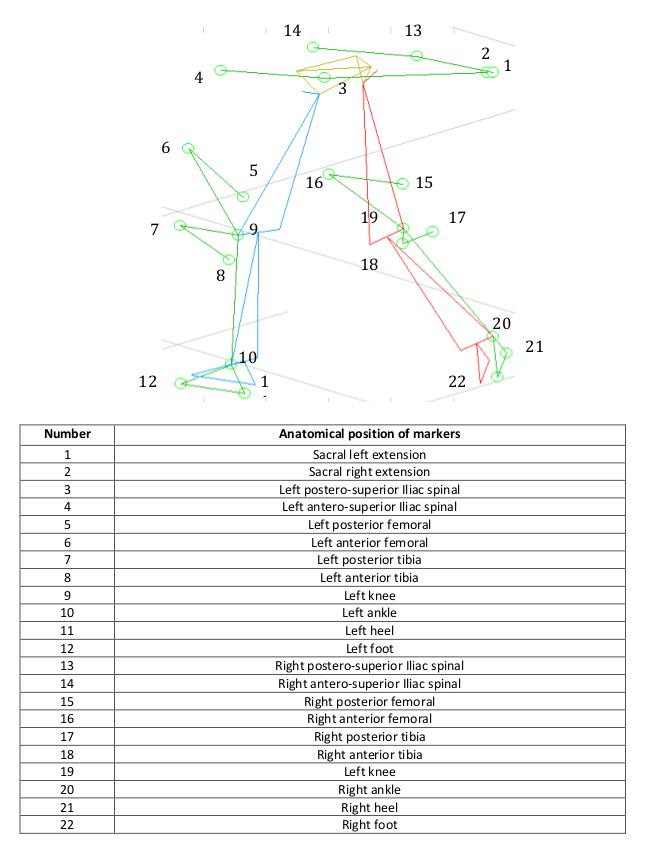

Supplement: S1 Fig — (TIF) [file pone.0192345.s004.tif]

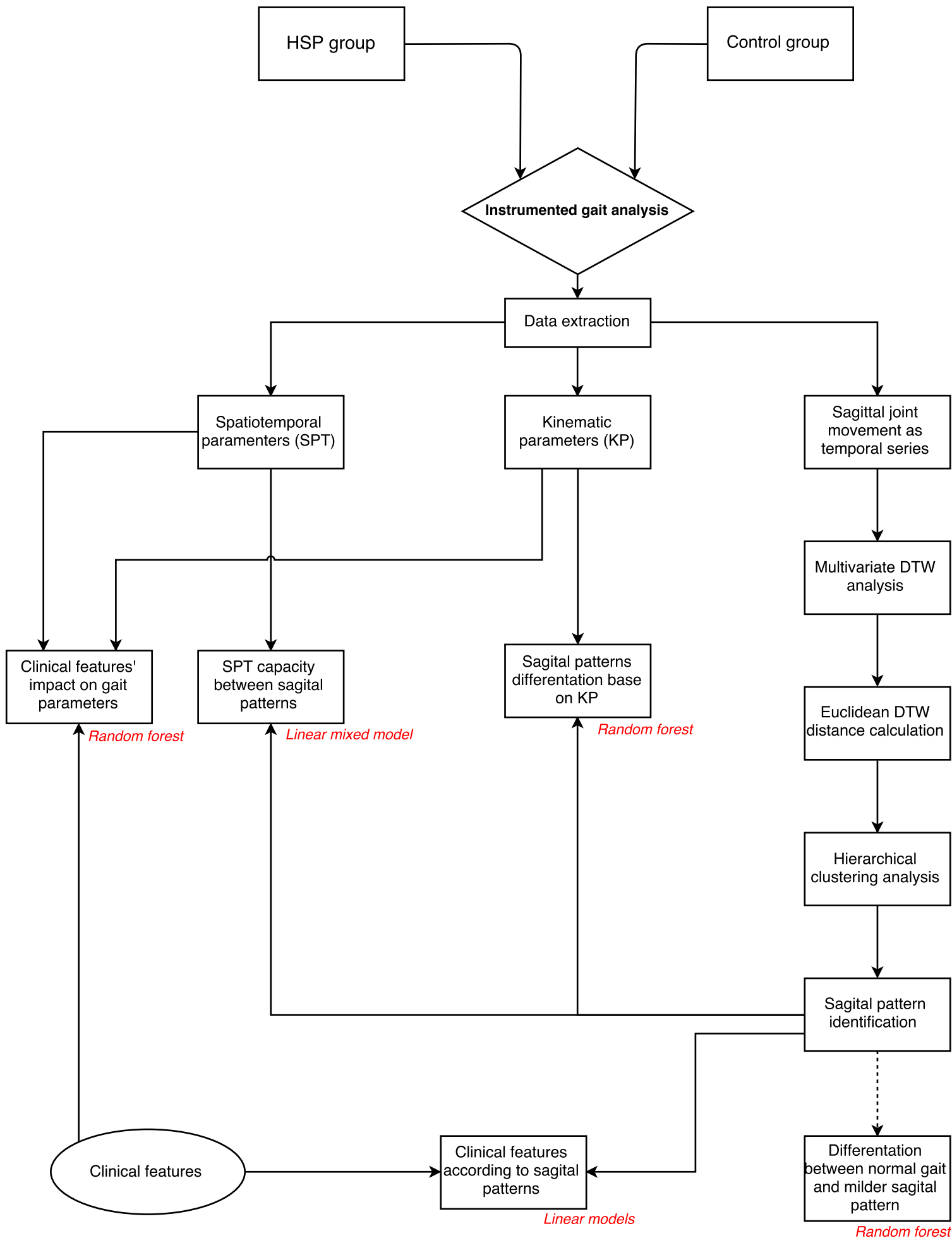

Supplement: S2 Fig — (PDF) [file pone.0192345.s005.pdf]

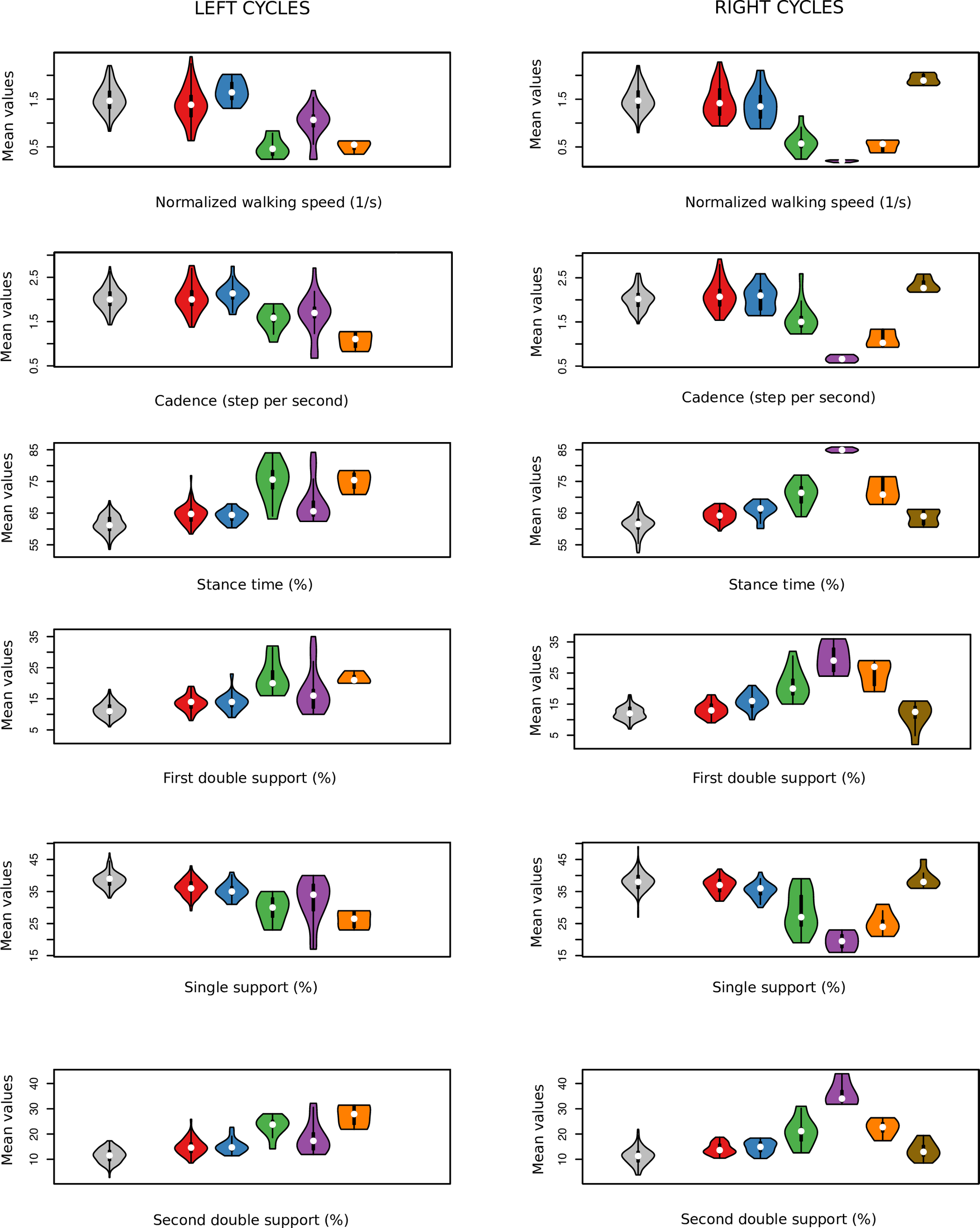

Supplement: S3 Fig — (Pattern I, in red; Pattern II, in blue; Pattern III, in purple; Pattern IV, in green; Pattern V, in orange; Pattern VI in brown.) and reference group (in grey). The graphs on the left show the results for left cycles; the graphs of the right show the results for right cycles. The vertical axis represents the value of the spatio-temporal parameters studied. The unit for each spatio-temporal parameter is summarized in Table 1. In each violin plot, the white point represents the average value of this pattern, the vertical black line represents the range. The shape of the violin plot depends on the distribution of the values of the spatio-temporal variable in the cycles classified in a particular pattern. (TIF) [file pone.0192345.s006.tif]

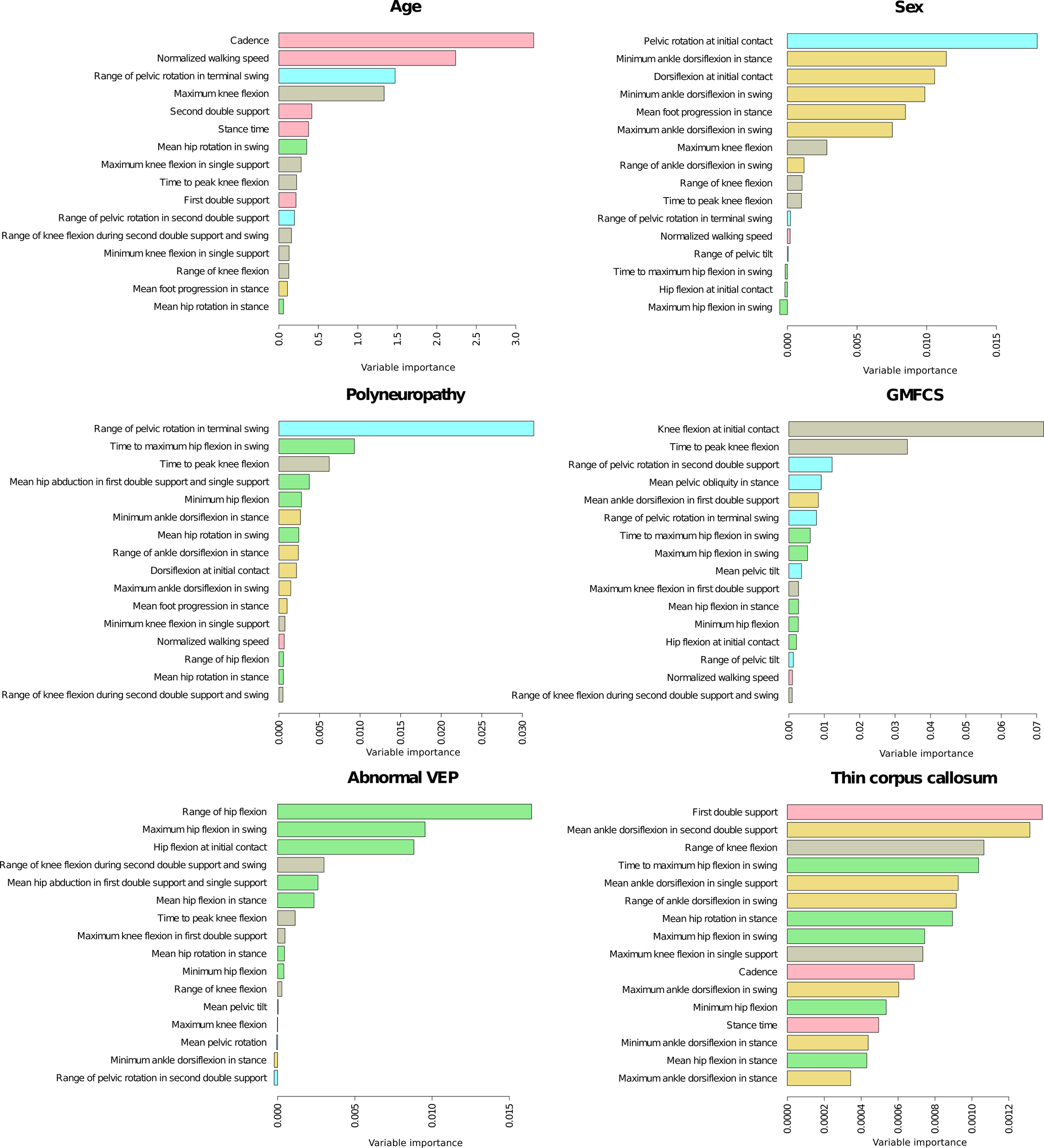

Supplement: S4 Fig — Importance is measured by Breiman-Cutler permutation variable importance (VIMP). Notice different scales in each random forest. Different colours indicate different joints (blue for pelvis, green for hip, brown for knee, and yellow for ankle parametes) and spatio-temporal parameters (pink). (TIF) [file pone.0192345.s007.tif]
